# Non-apical plateau potentials and persistent firing induced by metabotropic cholinergic modulation in layer 2/3 pyramidal cells in the rat prefrontal cortex

**DOI:** 10.1371/journal.pone.0314652

**Published:** 2024-12-10

**Authors:** Nicholas Hagger-Vaughan, Daniel Kolnier, Johan F. Storm

**Affiliations:** Brain Signalling Laboratory, Section for Physiology, Institute of Basic Medical Sciences, University of Oslo, Oslo, Norway; University of Nebraska Medical Center College of Medicine, UNITED STATES OF AMERICA

## Abstract

Here we describe a type of depolarising plateau potentials (PPs; sustained depolarisations outlasting the stimuli) in layer 2/3 pyramidal cells (L2/3PC) in rat prefrontal cortex (PFC) slices, using whole-cell somatic recordings. To our knowledge, this PP type has not been described before. In particular, unlike previously described plateau potentials that originate in the large apical dendrite of L5 cortical pyramidal neurons, these L2/3PC PPs are generated independently of the apical dendrite. Thus, surprisingly, these PPs persisted when the apical dendrite was cut off (~50 μm from the soma), and were sustained by local calcium application only to the somatic and basal dendritic compartments. The prefrontal L2/3PCs have been postulated to have a key role in consciousness, according to the Global Neuronal Workspace Theory: their long-range cortico-cortical connections provide the architecture required for the “global work-space", “ignition”, amplification, and sustained, reverberant activity, considered essential for conscious access. The PPs in L2/3PCs caused sustained spiking that profoundly altered the input-output relationships of these neurons, resembling the sustained activity suggested to underlie working memory and the mechanism underlying “behavioural time scale synaptic plasticity” in hippocampal pyramidal cells. The non-apical L2/3 PPs depended on metabotropic cholinergic (mAChR) or glutamatergic (mGluR) modulation, which is probably essential also for conscious brain states and experience, in both wakefulness and dreaming. Pharmacological tests indicated that the non-apical L2/3 PPs depend on transient receptor potential (TRP) cation channels, both TRPC4 and TRPC5, and require external calcium (Ca^2+^) and internal Ca^2+^ stores, but not voltage-gated Ca^2+^ channels, unlike Ca^2+^-dependent PPs in other cortical pyramidal neurons. These L2/3 non-apical plateau potentials may be involved in prefrontal functions, such as access consciousness, working memory, and executive functions such as planning, decision-making, and outcome prediction.

## Introduction

Plateau potentials (PPs) are long-lasting (typically > 100 ms) membrane potential depolarisations that are generated and sustained by intrinsic membrane properties even after the stimulus that triggered them has been terminated [1]. They can trigger persistent spike firing (PF) or enhance the probability of such firing that can continue for several seconds, sometimes requiring an inhibitory input to switch it off [2]. PPs have been observed in a variety of neuron types, and have been suggested to be involved in several functions, including working memory, neural plasticity, associative learning, and conscious perception [3–6]

There is considerable evidence for PPs or other intrinsically generated sustained depolarisations of dendritic origin, for example in the apical dendrites of neocortical pyramidal cells, generated by dendritic voltage-gated Ca^2+^ channels (VGCCs) or N-methyl-d-aspartate receptors (NMDARs), and resulting in somatic sustained spiking or bursts [7, 8]. PPs have been suggested as a mechanism for maintaining working memory [2, 4, 5], and shown to be important for induction of long-term potentiation (LTP) in apical dendritic synapses in vivo [3].

Apical dendritic calcium spikes and PPs have also been posited as a mechanism for integration of different information streams, which is needed for conscious sensory perception according to the dendritic integration theory of consciousness [6, 9]. Thus, in rodent layer 5 (L5) pyramidal cells, simultaneous synaptic input to the perisomatic region and the distal apical dendrite can cause a dendritic Ca^2+^ spike/PP that triggers an output of axonal/somatic bursts of Na^+^ action potentials [8]. The ‘apical amplification’ caused by coincident synaptic inputs from sensory (i.e. thalamic, perisomatic targeting) and contextual (cortico-cortical, apical targeting) information streams provide, per the theory, a key mechanism for conscious perception [6, 10, 11].

PPs and PF can often be induced or enhanced by neuromodulation, e.g. by acetylcholine (ACh) via muscarinic receptors (mAChRs; [12, 13], dopamine [14, 15], serotonin [16, 17], or via metabotropic glutamate receptors (mGluRs) [18]. Neuromodulator-induced PPs and PF have been found in a variety of excitatory neuron types, in several brain regions, including the hippocampus [19, 20] and neocortex [21].

The prefrontal cortex (PFC) is considered to be of key importance for executive functions, including planning [22], decision-making [23], and outcome prediction [24]. The PFC is also posited as being essential for consciousness in several leading theories, including the global neuronal workspace theory [25] and the higher-order theory of consciousness [26].

Layer 2/3 pyramidal cells (L2/3PCs) in the PFC have been postulated to have a key role in consciousness, in particular in the GNWT, where the long-range cortico-cortical connections formed by L2/3PCs provide the architecture required for the “global work-space" and its “ignition” and reverberant network activity, which is considered essential for conscious access [25, 26]. Whilst persistent network activity is likely dependent on recurrent synaptic connections [27], intrinsic cellular properties promoting enhanced and sustained firing are likely often necessary to support this network activity [28]. Thus, in a network model of the hippocampal CA3 network, persistent network activity was supported by a combination of synaptic changes and intracellular PP mechanism [28] induced by mAChR activation [29].

This suggests that networks can be enabled to support persistent, recurrent activity, which may underpin conscious processing and memory, by intrinsic cellular properties induced by neuromodulators. This is of particular interest in the case of ACh, which is released in the cortex at high levels during wakefulness and dreaming (REM) sleep when there are different forms of conscious experience, in contrast to the unconscious state of dreamless sleep, when the ACh release is far lower [30, 31].

In this study, we described and characterised ACh-induced plateau potentials (PPs) in PFC L2/3 pyramidal cells, and investigated the underlying mechanisms, including which ion channels generate the PPs and which subcellular compartments are needed for their generation.

## Methods

### Animals

All experimental procedures were approved by the responsible veterinarian of the Institute, in accordance with the statute regulating animal experimentation given by the Norwegian Ministry of Agriculture, 1996. For use in optogenetics experiments (see below, and **S5 Fig**), transgenic Cre reporter allele mice under the control of the cholinesterase gene (Chat-IRES-Cre; Stock No. 006410) were cross-bred with Ai32 mice (B6.Cg-Gt(ROSA)26Sortm32(CAG-COP4*H134R/EYFP)Hze/J; Stock No. 024109), thus generating experimental animals expressing yellow fluorescent protein (YFP) and channelrhodopsin in cholinergic cells. All transgenic animals were obtained from The Jackson Laboratory.

### Brain slice preparation

Young male Wistar rats (P21-P28) or adult (transgenic) mice of either sex, were briefly anaesthetised with suprane before decapitation and removal of the brain into ice-cold cutting solution containing (in mM): NaCl 87, KCl 1.25, KH_2_PO_4_ 1.25, NaHCO_3_ 25, glucose 16, sucrose 75, MgCl_2_ 7.0, CaCl_2_ 0.5. Coronal slices of 350–400 μm thickness were made in an ice-cold cutting solution using a VT1200 vibratome (Leica Microsystems, Wetzlar, Germany). The slices were transferred to a submerged holding chamber containing the artificial cerebrospinal fluid (aCSF) used for recording (described below) at 35°C for 30 minutes before being moved to room temperature (20–24°C). For experiments with cells with truncated apical dendrites (**Fig 6 and S5 Fig**), the slice was cut parallel to the pia approximately at the level of the layer 1/layer 2 border, using a scalpel blade immediately prior to recording.

### Electrophysiology

For most recordings slices were submerged in an aCSF containing (mM): NaCl 125, KCl 2.25, KH_2_PO_4_ 1.25, NaHCO_3_ 25, glucose 16, MgCl_2_ 1.5, CaCl_2_ 2.0, DNQX 10 μM, SR 95531 (gabazine) 5 μM, DL-2-amino-5-phosphonovalerate (AP5) 50 μM. For experiments using reduced-Ca^2+^ aCSF, the composition was as above except for the following changes (in mM): MgCl_2_ 4.0, CaCl_2_ 0.1, EGTA 0.2 (**Figs 4 and 5**). Cells were viewed using IR-DIC optics on a BX51WI microscope (Olympus, Tokyo, Japan). Whole-cell, somatic patch-clamp recordings were obtained from L2/3PCs in the paralimbic or infralimbic subregions of the prefrontal cortex. Patch pipettes were pulled from borosilicate glass using a Narashige PC-10 vertical puller (Narashige, Japan). Pipettes had tip resistances of 4–6 MΩ and were filled with a solution containing (in mM): K-methanesulphonate 120, HEPES 10, KCl 20, MgATP 4, NaGTP 0.4, Na_2_phosphocreatine 5, EGTA 1.0. The pH was adjusted to 7.3 and the osmolarity was adjusted to 290 mOsmol/l. For some experiments, Alexa 488 10 μM, Alexa 555 15 μM, or biocytin 0.3% was added to the pipette solution for tracing the cell morphology. The liquid junction potential was 9.4 mV and was not corrected for. Current-clamp recordings were performed using a Multiclamp 700A amplifier (Molecular Devices, Sunnyvale, CA, USA) or a Dagan BVC-700A (Dagan Corp, Dagan, Minneapolis, MN, USA). Local chemical application was achieved using a Picospritzer 2 (Parker, Hollis, NH, USA). Cells were filled with Alexa 555 for up to 30 minutes before fluorescence was observed using a mercury lamp and an Andor Zyla 4.2 sCMOS camera (Oxford Instruments, Abingdon, UK). This was then used to guide the pipette for drug application to the appropriate part of the cell. Same-sized pipettes were used for focal application as for whole-cell patch-clamp. Pipettes used for focal application contained modified aCSF, with the inclusion of (in μM): muscarine 10, Alexa 555 10, DNQX 10, gabazine 5, and AP5 50. For experiments using reduced-Ca^2+^, the following changes were made: 5 mM Ca^2+^, 0.5 mM Mg^2+^. In experiments with focal application of FFA, 200 μM FFA (in DMSO, final concentration in extracellular aCSF was >0.1%) was added. In control experiments, DMSO was used instead of FFA. A 60-s long pressure pulse of 1–10 psi, with the pipette 10–40 μm from the soma, or near the first bifurcation of the apical dendrite, was used, starting 30 seconds before the induced spike-train. For experiments with focal application of FFA and the corresponding control experiments, a 120-s long pulse was used instead, starting 90 seconds before the induced spike-train.

### Optogenetics

Activation of cholinergic afferents occurred by a train of 5 or 10 blue light (λ = 460–480) pulses, 10 ms long, at 25 Hz, from a mercury lamp (Olympus, Tokyo, Japan) or a xenon lamp (Lambda XL, Sutter, Novato, CA, USA) (**S5 Fig**). Illumination was centred on the soma using either a 40x (NA 0.8) or a 60x (NA 0.9) immersion lens. In all experiments with photoactivation of cholinergic afferents, 10 μM gabazine and 10 μM physostigmine were included in the aCSF.

### Data acquisition and analysis

Data were acquired using pClamp 10.7 software, and the data were digitised using a Digidata 1440A (Molecular Devices). Analysis was performed in Clampfit 10.7 (Molecular Devices), and plotted in Origin 9.7 (OriginLab, Northampton, USA).

### Histology

Cells intended for staining and reconstruction were filled with biocytin during electrophysiological recording and fixed in 4% paraformaldehyde in phosphate-buffered saline overnight at 4°C, then transferred into phosphate-buffered saline at 4°C for up to two weeks. The avidin-biotin-peroxidase method was used to visualise the cells, using 3,3′-diaminobenzidine as a chromogen (ABC kit from Vector Laboratories, Burlingame, CA, USA) and morphological reconstruction was performed using the Neurolucida system (MicroBrightField, Colchester, VT, USA).

### Chemicals

DNQX, gabazine, DL-AP5, ML 204, AC 1903, nifedipine, muscarine, tACPD and physostigmine were acquired from Tocris. All chemicals were bath applied at a superfusion rate of ~2 ml min^−1^, except where described otherwise. Note that in experiments with bath-applications of drugs/chemicals or modified aCSF (e.g. in **Figs 2–4**), we normally compared the changes seen after the bath-application (i.e. after a time giving apparently full effect; typically after a few minutes) with changes seen in time-matched control recordings (usually in different cells), when no chemical was applied and the aCSF was not changed. This is a more informative comparison than simply comparing parameters before and after each application, because several parameters can change spontaneously over time, even when the aCSF was not changed.

### Statistics

Statistical analysis was performed with GraphPad Prism, version 9. Grouped data are expressed as mean ± SEM, and the sample size of cells (*n*). Non-parametric tests were used to determine statistical significance as sample sizes were too small to accurately determine whether they were normally distributed. Paired data were checked for significance using the Wilcoxon Signed-Rank (WSR) test, whilst the Mann-Whitney U (MWU) test was used for unpaired data. Summary graph plots include all data points as well as mean lines and 95% confidence intervals (CI) represented by whiskers. The post-burst depolarisation (PBD) was defined as the mean membrane potential 10 seconds after the last spike of the induced spike-train relative to the membrane potential before the initiation of the spike-train. The PBD and spike data used in summary graphs represent values obtained immediately before and 5 minutes after the application of the compound in question. For some figures, we analysed the change in PBD (Δpost-burst depolarisation) and in spikes (Δspikes) from before to after application, comparing this change between the experimental groups and a time-matched control, resulting in some cases in negative PBD and negative spiking, as the change was smaller than in the control. The time-matched control refers to values from control cells that were sampled at identical time points as the experimental group, ensuring that muscarine had been present in the bath for the same amount of time and elicited the same amount of plateau potentials. This was done to compensate for any time- or use-dependant changes to the muscarinic plateau potentials. Differences between groups were considered significant at *p* < 0.05 and are denoted in graphs by an asterisk. Group differences with *p* > 0.05 were considered non-significant and are denoted in graphs by “ns”.

## Results

### mAChR-dependent plateau potentials in PFC L2/3PCs

**Fig 1:**
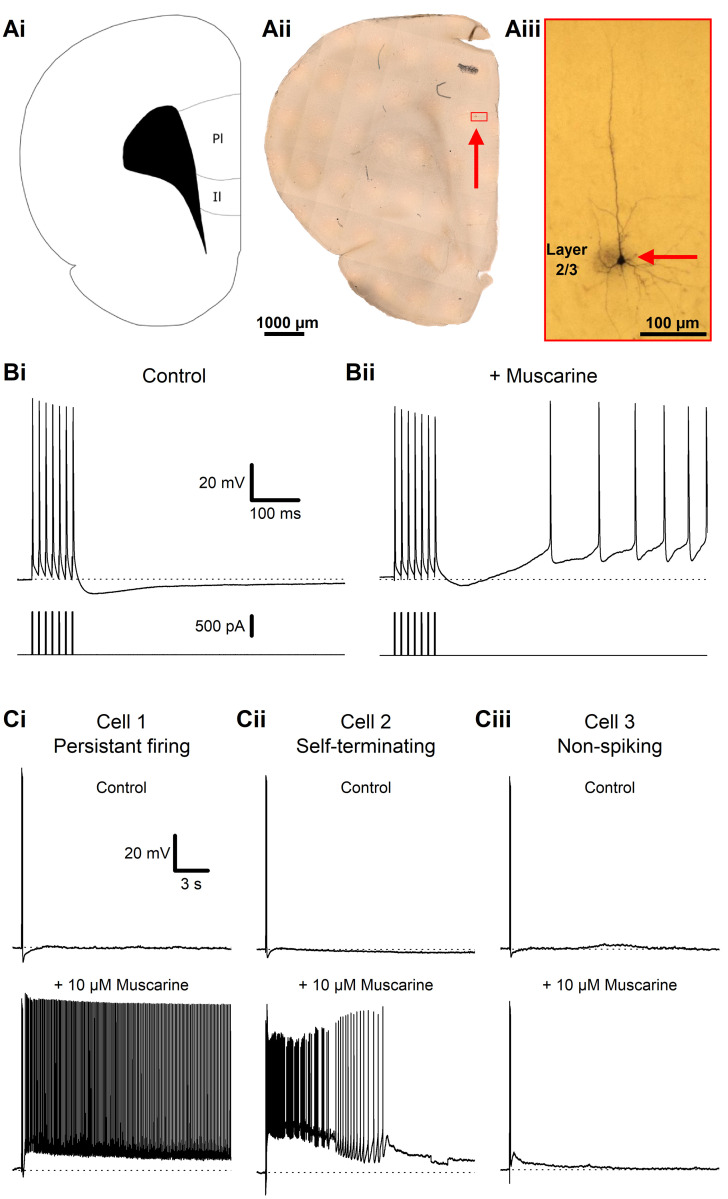
Muscarinic plateau potentials in L2/3PCs of the PFC. **A**—Visual overview over an example recorded cell, with a schematic of a coronal brain slice (i) showing the PFC regions where cells were recorded from [32], a fixed rat PFC slice (ii), shown at low magnification, with a red box marking the location of the biocytin stained L2/3PC seen in (iii). **B**—Somatic whole-cell recording traces from an L2/3PC, showing membrane potential responses to a train of 7 action potentials at 70 Hz (top traces), evoked by injected brief depolarising current pulses (bottom traces), under control conditions (i), and after the bath-application of 10 μM muscarine (ii). **C—**Example traces of different membrane potential responses in three different cells (i-iii) after a train of current pulses during control condition (top traces) and after the bath application of 10 μM muscarine (bottom traces).

We obtained stable whole-cell recordings from 102 layer 2/3 pyramidal cells from the prelimbic and infralimbic regions of the prefrontal cortex in acute brain slices from rats and mice (**Fig 1A and 1B**). To elicit spike trains, we used depolarising somatic injections of 7 brief (2 ms) depolarising current (1–1.5 nA) pulses at 70 Hz, each triggering a single action potential (hereafter referred to as the spike-train protocol).

In control conditions (normal aCSF) each spike train was followed by a slow after-hyperpolarisation (AHP; **Fig 1Bi**), lasting several hundred milliseconds, but during bath-application of 10 μM muscarine, the spike train elicited a range of depolarising changes in the membrane potential, which varied between cells (**Fig 1C**): from sustained PPs, eliciting spike firing for several seconds (**Fig 1Ci**) to briefer depolarisations with (**Fig 1Cii**) or without (**Fig 1Ciii**) spiking. Here, both long-lasting PPs and briefer post-burst depolarisations (PBDs) will be referred to as PPs, since they seem to represent different degrees of the same phenomenon, and share key properties and mechanisms (see below).

**Fig 2:**
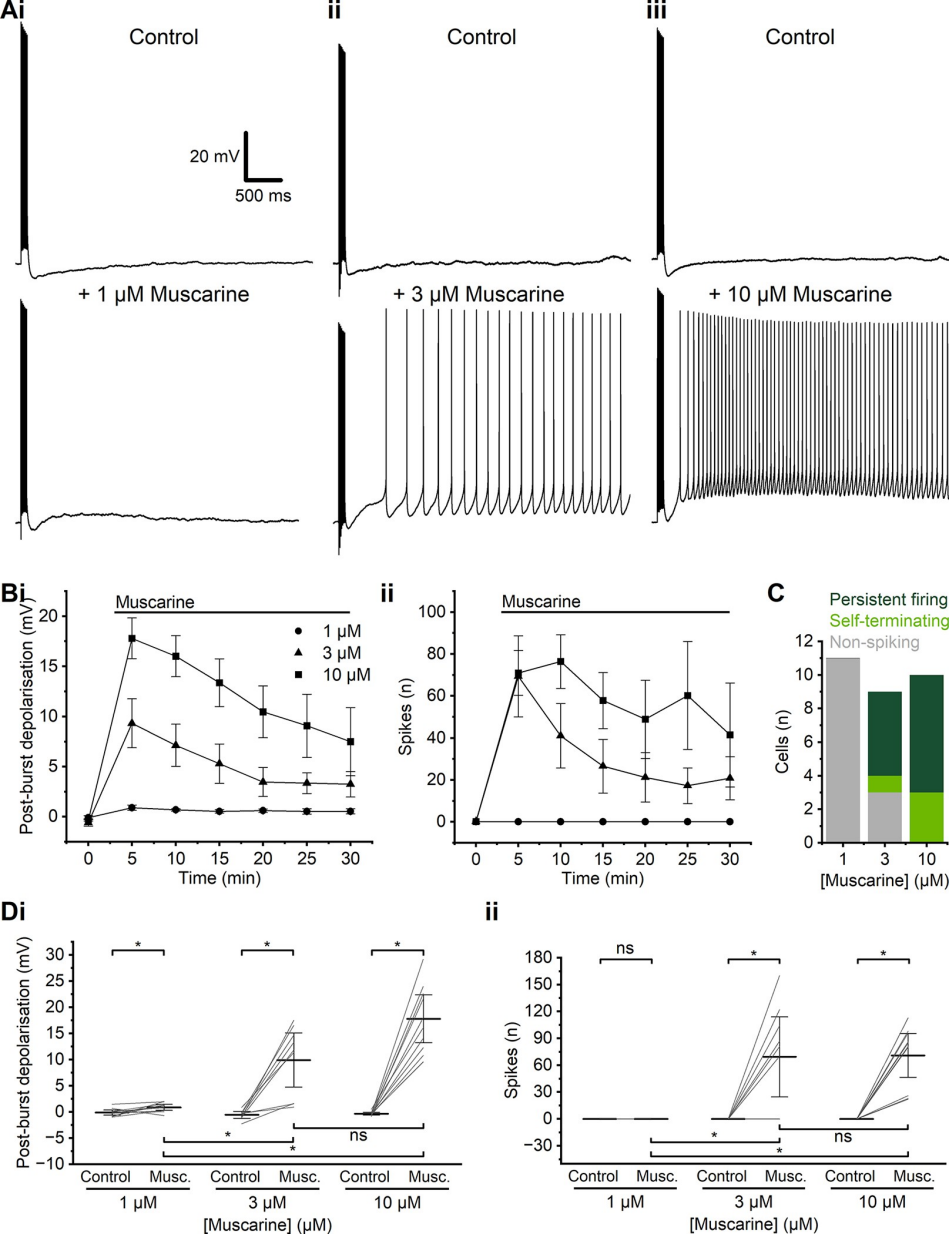
Plateau potentials depended on the concentration of muscarine. **A**—Example traces showing characteristic responses following an action potential train during the application of 1 μM (i), 3 μM (ii), or 10 μM muscarine (iii). **B**—Time course comparison of PBD (i) and spikes (ii) at different muscarine concentrations. **C**—Summary graph of the incidence of persistent firing, self-terminating and non-spiking evoked by different muscarine concentrations. **D**—Summary graphs for the PBD (i) and spikes (ii) evoked by different muscarine concentrations. (i) Data shows a significant increase in PBD at all concentrations tested of muscarine compared to control conditions. (WSR test, 1 μM: *p* = 0.02, *n* = 10; 3 μM: *p* = 0.03, *n* = 9; 10 μM: *p* < 0.01, *n* = 10). Comparison of PBD between different concentrations of muscarine shows a significantly larger PBD at 3 and 10 μM compared to 1 μM muscarine, but no significant difference between 3 μM and 10 μM (MWU test, 1 μM vs 3 μM: *p* < 0.01; 1 μM vs 10 μM: *p* < 0.01; 3 μM vs 10 μM: *p* > 0.05). (ii) Summary graph of the incidence of spiking during PPs elicited following the application of different muscarine concentrations. A Ssignificant increase in the incidence of spikes during PPs in 3 and 10 μM muscarine compared to control conditions was observed, but no incidents of spiking PPs with 1 μM muscarine. (WSR test, 1 μM: p = not applicable, *n* = 10; 3 μM: *p* = 0.03, *n* = 9; 10 μM: *p* < 0.01, *n* = 10). Comparison of spiking during PPs shows a significant difference in spiking between both 3 and 10 μM compared to 1 μM, but no significant difference in spiking between 3 μM and 10 μM muscarine (MWU test, 1 μM vs 3 μM: *p* < 0.01; 1 μM vs 10 μM: *p* < 0.01; 3 μM vs 10 μM: *p* = 0.92). Continuous horizontal bars above the time-course plots indicate the extracellular presence in aCSF of the stated compounds in this and all following figures.

We tested how PPs depend on the concentration of muscarine (**Fig 2**). The lowest muscarine dose (1 μM) abolished the slow AHP but elicited only a small PBD (**Fig 2Ai**) (control: -0.10 ± 0.21 mV; 1 μM muscarine: 0.90 ± 0.25 mV, *n* = 10, *p* = 0.02), which did not trigger any spikes in the ten cells tested. (Note that the values for changes in membrane potential (PBD) were measured 5 minutes after the onset of muscarine application, and are always compared to time-matched values for control recordings, to compensate for any time-dependent changes. The PBD values may thus occasionally be negative.) Tripling the muscarine concentration (3 μM) induced a stronger PBD a (control: -0.56 ± 0.29 mV; 3 μM muscarine: 9.9 ± 2.2 mV, *n* = 9, *p* < 0.01), which in most cells triggered spiking (control: 0 ± 0 spikes; 3 μM muscarine: 69 ± 19 spikes, *n* = 9, *p* = 0.03). Increasing the muscarine concentration to 10 μM gave a still larger PBD (control: -0.36 ± 0.12 mV; 10 μM muscarine: 18 ± 2.0 mV, *n* = 10, *p* < 0.01) and more spiking (control: 0 ± 0 spikes; 10 μM muscarine: 71 ± 11 spikes, *n* = 10, *p* < 0.01) (**Fig 2B and 2C**). The values compared in **Fig 2D** were measured after 5 minutes of drug application (or no drug, for the controls). The different muscarine concentrations (1, 3, and 10 μM) were tested in different cell groups, not added cumulatively in the same cells.

**Fig 3:**
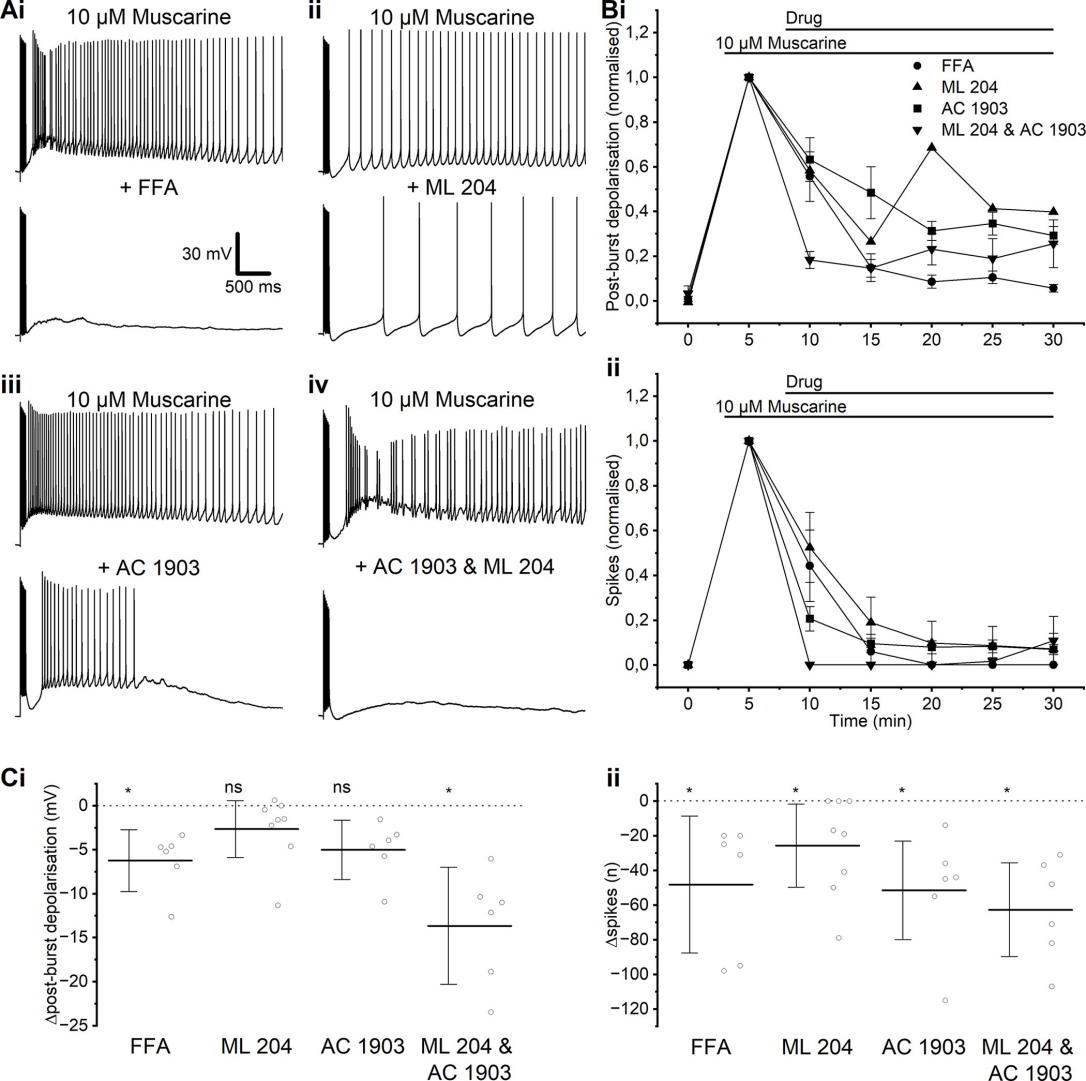
Evidence that plateau potentials depend on TRPC channels. **A**—Example traces showing the muscarinic PP before and after the application of TRP-blocking drugs FFA (i), ML 204 (ii), AC 1903 (iii), and ML 204 & AC 1903 (iv). **B**—Time course comparison of PBD (i) and spikes (ii) with different TRPC channel-blocking drugs. **C**—Summary plots of the PBD (i) and spikes (ii) before and after the application of different TRPC channel-blocking drugs. Significance was calculated in comparison to time-matched control measures. (i) A significant change in PBD was observed after the wash-in of FFA and ML 204 & AC 1903. (MWU test, FFA: *p* = 0.02, *n* = 6; ML 204 & AC 1903: *p* < 0.01, *n* = 6). There was no significant change in PBD when ML 204 or AC 1903 was washed in individually. (MWU test, ML 204; *p* = 0.83, *n* = 8; AC 1903: *p* = 0.11, *n* = 6). (ii) A significant change in spike count during PPs was observed in all groups compared with controls. (MWU test, FFA: *p* = 0.01, *n* = 6; ML 204: *p* = 0.04, *n* = 8; AC 1903: *p* < 0.01, *n* = 6; ML 204 & AC 1903: *p* < 0.01, *n* = 6).

**Fig 4:**
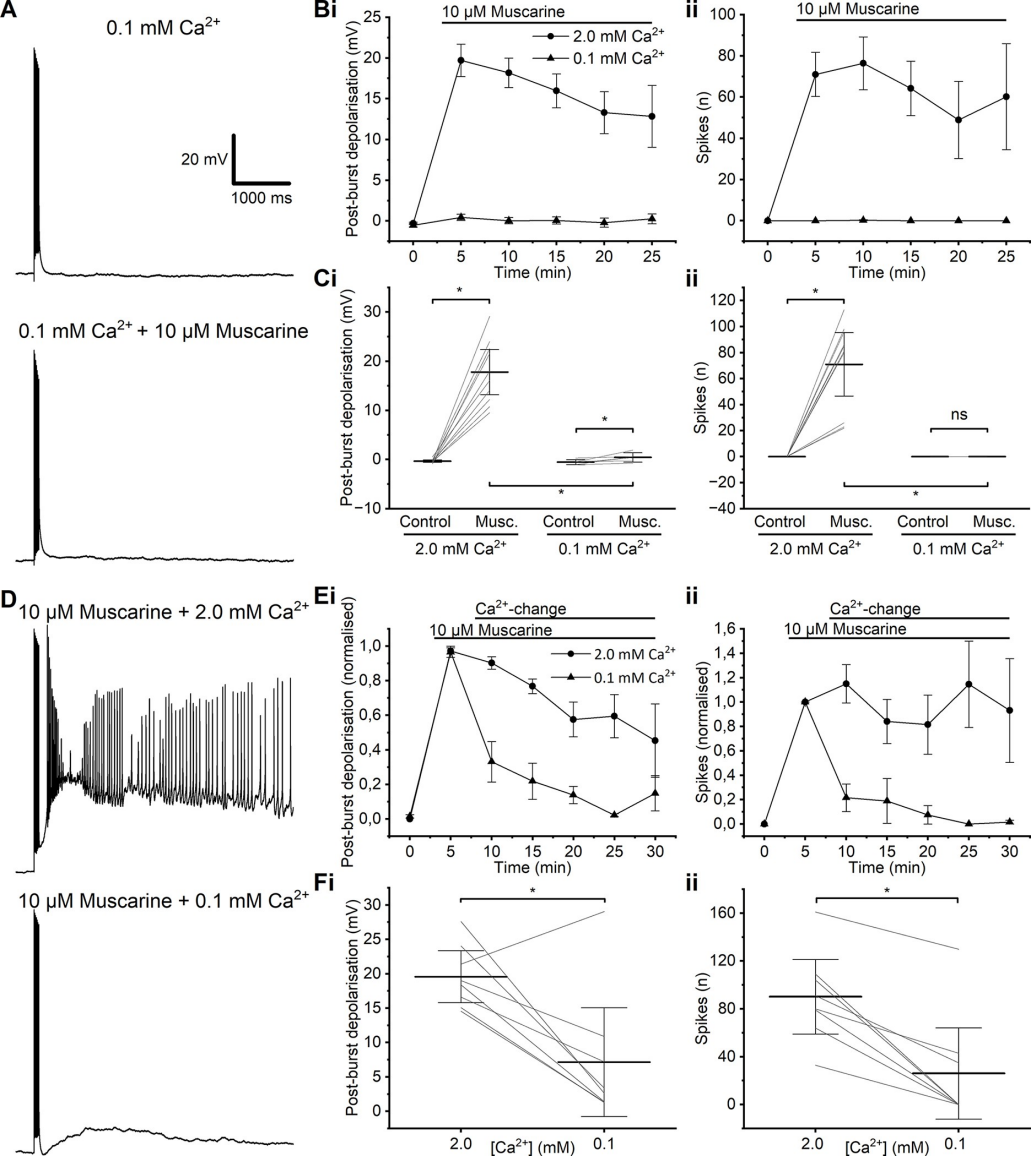
The plateau potential is Ca^2+^-dependent. **A**—Example traces of the effect of adding muscarine to low-Ca^2+^ aCSF, and the absence of an evoked PP. **B**—Time course summary of PBD (i) and spikes (ii) evoked by muscarine in normal- or low-Ca^2+^ aCSF. **C**—Summary graphs showing the effect of muscarine on PBD (i) and spikes (ii) in either normal- or low-Ca^2+^ aCSF. (i) Wash-in of 10 μM muscarine in both normal- and low-Ca^2+^ aCSF resulted in a significant change in PBD. (MWU test, 2.0 mM Ca^2+^: *p* < 0.01, *n* = 10; 0.1 mM Ca^2+^: *p* = 0.03, *n* = 6). (ii) A significant change in post-burst spiking was observed after the wash-in of muscarine in normal-high Ca^2+^, but no spiking PPs in low- Ca^2+^. (MWU test, 2.0 mM Ca^2+^: *p* < 0.01, *n* = 10; 0.1 mM Ca^2+^: *p* = not applicable, *n* = 6). There was a significant difference in both PBD and spike number between muscarine trials in normal- and low-Ca^2+^ aCSF (MWU test, *n* = 6, PBD: *p* = 0.02; spikes: *p* < 0.01). **D**—Example traces showing the effect of switching from normal- to low-Ca^2+^ aCSF on a muscarinic PP. **E**—Time course summary of PBD (i) and spikes (ii) evoked by muscarine and the subsequent change when switching from normal- to low-Ca^2+^ aCSF. Presented alongside control data with no change in Ca^2+^. **F**—Summary graphs showing the effect of switching from normal- to low-Ca^2+^ aCSF on PBD (i) and spikes (ii) in the presence of muscarine. A significant change in both PBD and spikes was observed after switching from a normal-Ca^2+^ aCSF to a low-Ca^2+^ aCSF (WSR, *n* = 8).

**Fig 5:**
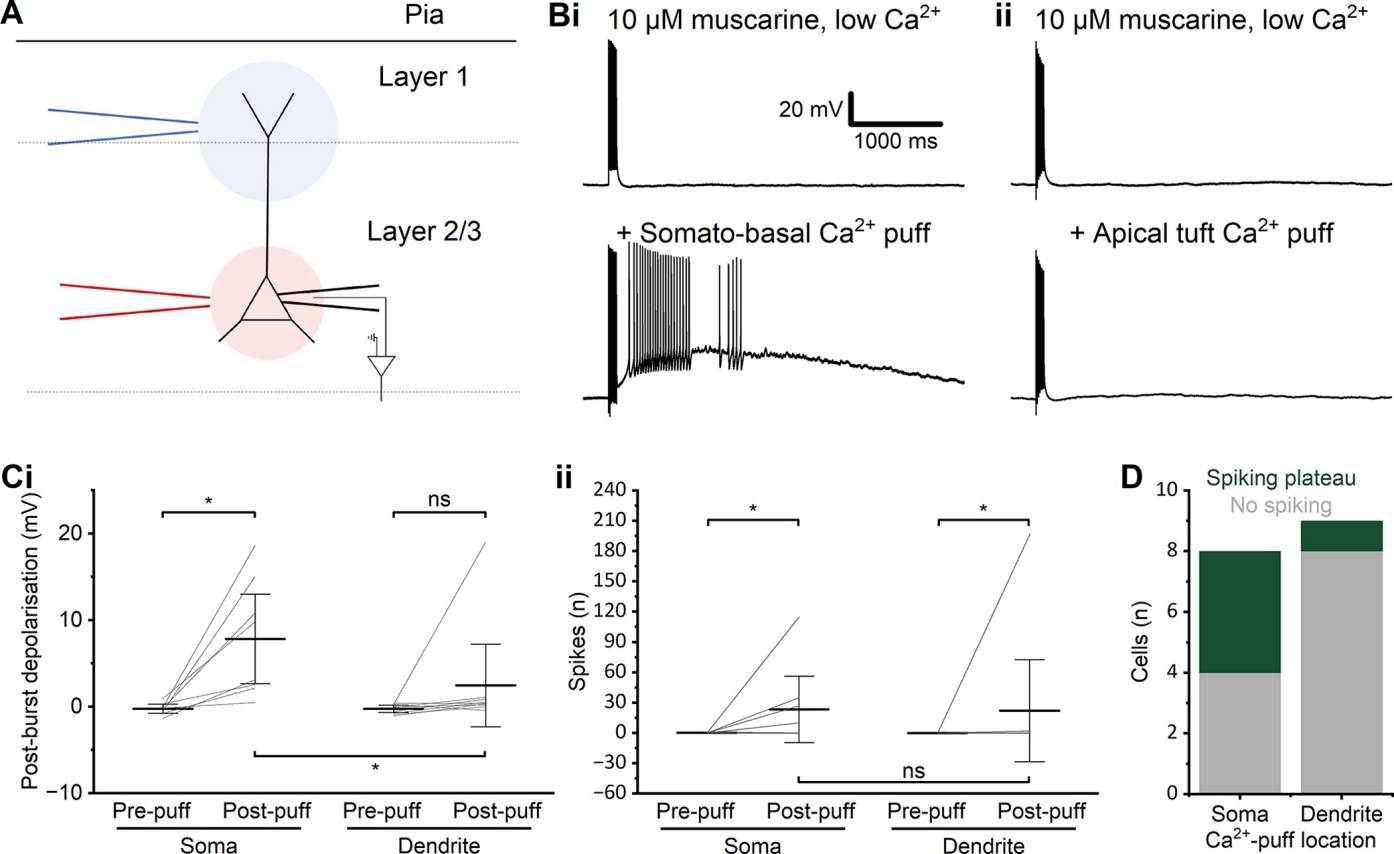
Peri-somatic/basal Ca^2+^ entry is essential for plateau potentials. **A**—Schematic representation of the experimental set-up for application of local Ca^2+^ either close to the soma (red) or apical dendrite (blue). **B**—Example traces showing the effect of local Ca^2+^ application either close to the soma (i) or apical dendrite (ii) in low-Ca^2+^ aCSF containing muscarine. **C**—Summary graphs showing the PBD (i) and spikes (ii) before and after calcium puffing at either the soma or dendrite. (i) A significant change in the PBD was observed after focal application of Ca^2+^ to the perisomatic region and, but not after application to the apical dendrite (WSR, soma: *p* < 0.01, *n* = 8; dendrite: *p* = 0.02, *n* = 9). The post-puff PBD was significantly larger after the application of Ca^2+^ to the perisomatic region than after application to the dendrite (*p* = 0.0218 by MWU). (ii) Post-burst spiking, however, did not change significantly in either application to the perisomatic region or to the dendrite (WSR, soma: *p* = 0.13, *n* = 8; dendrite: *p* = 0.50, *n* = 9). There was no significant difference between perisomatic and dendritic application, largely due to one outlier cell that spiked following dendritic application (*p* = 0.22 by MWU). **D**—Summary graph of the incidence of spiking vs. non-spiking PPs evoked by different locations of Ca^2+^ application.

**Fig 6:**
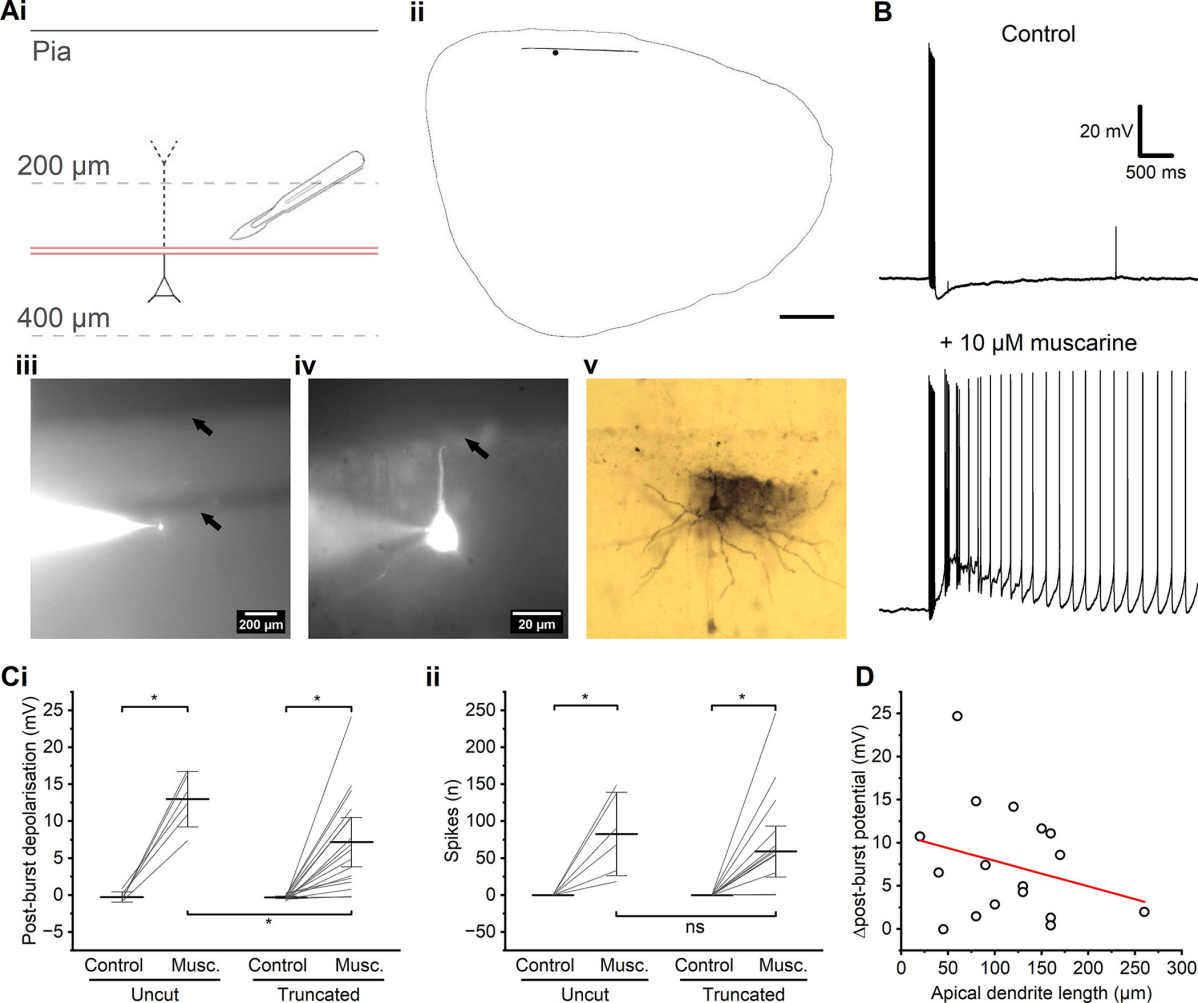
Plateau potentials can be elicited in the absence of the apical trunk. **A**—(i) Schematic illustration of experimental procedure for slice cutting, with the two red lines illustrating the cut, and the dotted lines illustrating the disconnected distal apical dendrite. (ii) Neurolucida tTracing of a coronal prefrontal rat brain slice, with thea cut detailed in (i) (illustrated by a black line), and a L2/3PC (represented by a black dot). (iii) A low magnification photomicrograph of the same cell illustrated in (ii), filled with Alexa 555. The top black arrow indicates the pia, while the bottom the black arrow indicates the cut. (iv) A high magnification photomicrograph of the same cell as seen in (iii and iv), with a black arrow indicating the cut. (v) A photomicrograph of the same cell as in (iv) following filling with and staining for biocytin. **B**—Example traces of a muscarinic PP evoked in a cell with a truncated apical dendrite. **C**—Summary plots comparing the PBD (i) and spikes (ii) in cells with truncated and intact apical dendrites. (i) A significant change in PBD was observed after the wash-in of muscarine in both the control and truncated groups (WSR test, truncated dendrite; *n* = 17, control; *n* = 6). There was also a significant difference in PBD between the truncated and control groups after the wash-in of muscarine (*p* = 0.024 by MWU test). (ii) A significant change in spikes was also observed after the wash-in of muscarine in both groups (WSR test, truncated dendrite; *n* = 17, control; *n* = 6). No significant difference in spikes was observed between the two groups after the wash-in of muscarine (*p* = 0.19 by MWU test). **D**—Summary plot of change in PBD following muscarine application against measured remaining apical dendrite length post-cut. (Spearman’s ρ = -0.18; *n* = 17; *p* = 0.48).

**Fig 7:**
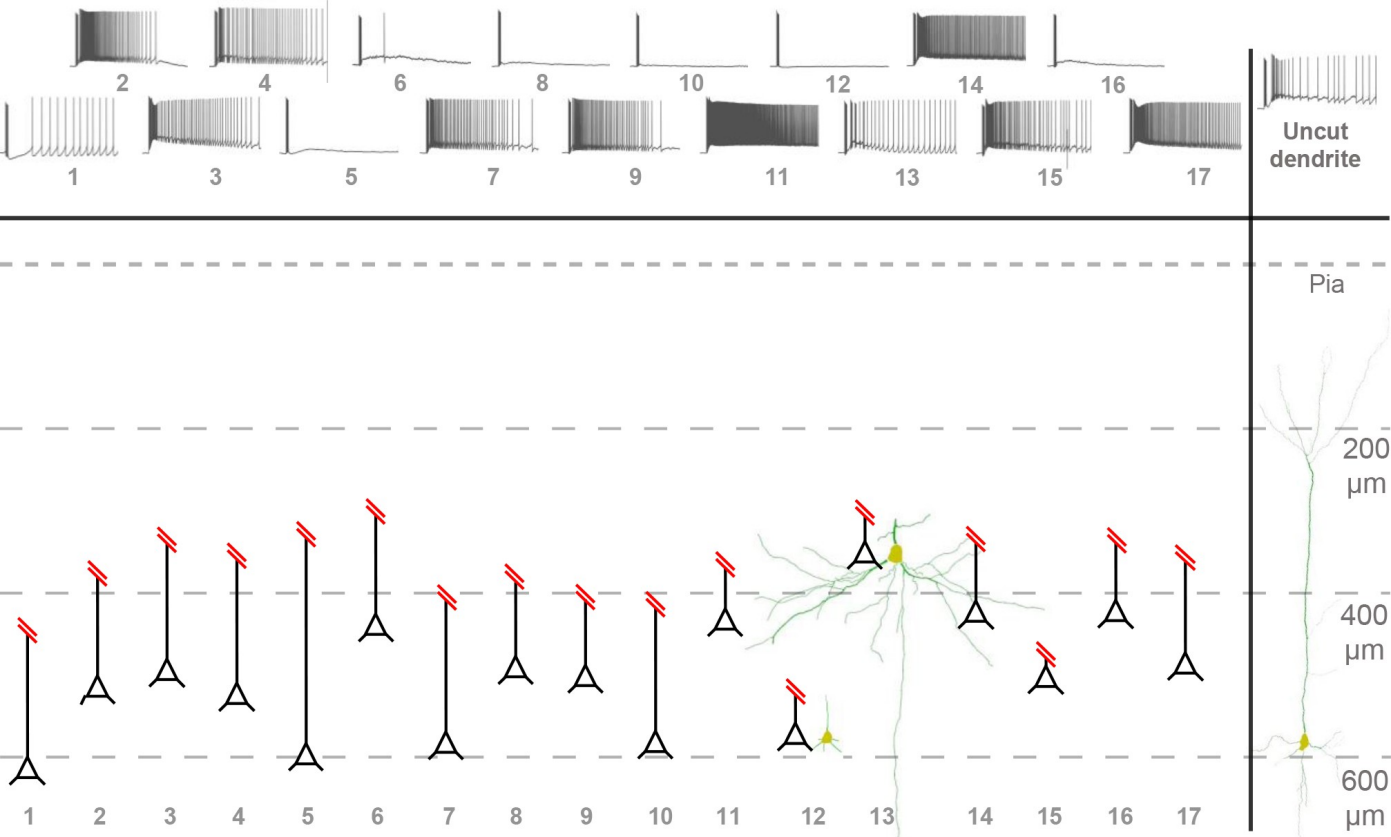
Overview of cells with truncated apical dendrites. Overview of cell morphologies post-cut as described in Fig 6. Schematic representations of cells represent soma distance from the pia mater (Pia) and intact apical dendrite trunk. Two reconstructed morphologies of truncated cells and one of a control cell are also included for further context. Example traces after the wash-in of 10 μM muscarine are included in the top row with cell numbering.

Muscarine elicited spiking PPs most consistently at 10 μM (**Fig 2D**; 10/10 cells) compared to 3 μM (6/9 cells) and 1 μM (0/11 cells). For this reason, we used 10 μM muscarine as the standard concentration to elicit PPs in all the following experiments (**Figs 3–7, S2–S5 Figs**). During long-lasting muscarine applications, the induced depolarisation (PBD) and spiking declined over time, but declined less with 10 μM than with 3 μM (**Fig 2B**).

Whilst these PPs were clearly mAChR-dependent, PPs have been observed elsewhere to be elicited following activation of several different metabotropic receptor types [17, 19, 33]. To test whether metabotropic glutamate receptors (mGluRs) also can induce PPs in L2/3PCs, we applied tACPD, a selective agonist of the mGluR group I/II GluRs. After application of 15 μM tACPD, the spike-train protocol elicited a robust and reliable PP (**S1A Fig**), with a significant PBD (control: -0.41 ± 0.10 mV; tACPD: 4.6 ± 1.1 mV, *n* = 12, *p* < 0.01) (**S1Bi Fig**) and spikes (control: 0 ± 0 spikes; tACPD: 23 ± 7.4 spikes, *n* = 12, *p* < 0.01) (**S1Bii Fig**). Since mAChRs and mGluRs are known to trigger similar signalling cascades, our results suggest that similar mechanisms of PP generation may be induced by a range or combination of different neuromodulators.

### L2/3PC PPs were dependent on TRPC channels

PPs have been shown to be dependent on transient receptor potential (TRP) channels in a variety of cell types [19, 34], and TRP channel activity is known to be enhanced by mAChR activation (Yoshida et al., 2012). Using the non-specific TRP channel blocker flufenamic acid (FFA; 50 μM) following the induction of PPs with muscarine, we observed a strong reduction in the PP relative to the time-dependant run-down in the control both in PBD (control: -1.8 ± 0.96 mV, *n* = 10; FFA: -6.2 ± 1.4 mV, *n* = 6, *p* = 0.02) and spikes (control: 5.4 ± 9.8 spikes, *n* = 10; FFA: -48 ± 15 spikes, *n* = 6, *p* = 0.01) (**Fig 3Ai, 3B and 3C**).

Previous work has identified the TRP channel subtypes TRPC4 and TRPC5 as being particularly abundant in the PFC (Fowler et al., 2007). We therefore used specific blockers for these channel subtypes to test if they were responsible for the muscarinic PP in L2/3PCs. Both the TRPC4 blocker ML 204 (10 μM) and the TRPC5 blocker AC 1903 (30 μM) produced a partial block of the PP (**Fig 3Aii and 3Aiii**), whilst the simultaneous application of both these blockers strongly reduced the PP (**Fig 3Aiv**). This suggests that both TRPC4 and TRPC5 are present in these cells and are involved in the generation of muscarine-induced PPs.

The change in mean PBD amplitude that we observed with the TRPC4 blocker ML 204 alone was not statistically significant compared to time-matched control experiments (control: -1.8 ± 0.96 mV, *n* = 10; ML 204: -2.6 ± 1.4 mV, *n* = 8, *p* = 0.83) (**Fig 3Ci**), but the spike number change relative to the control was significant (control: 5.4 ± 9.8 spikes, *n* = 10; ML 204: -26 ± 10 spikes, *n* = 8, *p* = 0.04) (**Fig 3Cii**). Also the TRPC5 blocker AC 1903 application gave a non-significant decrease in PBD (control: -1.8 ± 0.96 mV, *n* = 10, AC 1903: 5.0 ± 1.3 mV, *n* = 6, *p* = 0.12) (**Fig 3Ci**), but a significant decrease in spikes (control: 5.4 ± 9.8 spikes, *n* = 10; AC 1903: -52 ± 14 spikes, *n* = 6, *p* < 0.01) (**Fig 3Cii**).

The combination of ML 204 and AC 1903 gave the largest blocking effect on both change in PBD (control: -1.8 ± 0.96 mV, *n* = 10; ML 204 & AC 1903: -13.6 ± 2.6 mV (*n* = 6), *p* < 0.01) (**Fig 3Ci**) and post-burst spiking (control: 5.4 ± 9.8 spikes, *n* = 10; ML 204 & AC 1903: -63 ± 12 spikes, *n* = 6, *p* = 0.002 (**Fig 3Cii**).

Previous work has shown that TRPC channel modulation alters the impact of proximal apical EPSPs on PPs, but not the impact of distal apical EPSPs, and that FFA can be applied locally to probe the subcellular location of these channels [35]. We attempted to determine the subcellular location of the PP-generating channels in L2/3PCs, by local pressure application of FFA-containing aCSF from a glass pipette directed either towards the perisomatic region or distal apical dendrite, after PPs had been induced by bath-application of muscarine (**S2 Fig**). The PBD and the evoked spike number were clearly reduced in some cells following local application of FFA aimed at the perisomatic region, but not when applying FFA towards the dendrite (**S2AI and S2Aii Fig**). There were also small changes in the mean values of these parameters across all cells tested only for FFA-applications towards the perisomatic region, but not towards the apical dendrite (**S2Bi and S2Bii Fig**). However, the results varied considerably between cells, and neither the changes in PBD (control: -5.7 ± 1.5 mV, *n* = 6; FFA: -5.9 ± 0.58 mV, *n* = 10, *p* > 0.99) nor spikes (control: -30 ± 8.7 spikes, *n* = 6; FFA: -45 ± 12.4 spikes, *n* = 10, *p* = 0.45) were found to be statistically significant across all the tested cells.

### L2/3PC PPs were abolished by removal of extracellular calcium, but not by application of voltage-gated calcium channel (VGCC) blockers

To investigate whether the PPs are calcium-dependent [36, 37], we tested the generation of PPs in aCSF with a low concentration of free Ca^2+^, containing 0.1 mM Ca^2+^ and 0.2 mM EGTA. In this condition, the addition of muscarine did not induce spiking PPs (**Fig 4A**): there was no significant change in post-burst spiking (control: 0 ± 0 spikes, muscarine; 0 ± 0 spikes, *n* = 6) (**Fig 4Cii**), but a small, significant PBD (control: -0.57 ± 0.20 mV, *n* = 6; muscarine: 0.44 ± 0.37 mV, *n* = 6, *p* = 0.03) (**Fig 4Ci**).

In normal aCSF containing 2.0 mM Ca^2+^, PPs were induced by muscarine, but switching to muscarine-containing, low-Ca^2+^ aCSF abolished the PPs (**Fig 4D**). The decrease in PBD (normal-Ca^2+^: 19.6 ± 1.6 mV; low-Ca^2+^: 7.2 ± 3.4 mV, *n* = 8, *p* = 0.02) and spikes (normal-Ca^2+^: 90 ± 13 spikes; low-Ca^2+^: 26 ± 16 spikes, *n* = 8, *p* = 0.01) after switching to low-Ca^2+^ aCSF was significantly different (PBD: *p* < 0.01; spikes: *p* < 0.01) from the changes seen in time-matched control cells when the aCSF was not changed during the experiment (**Fig 4Fi and 4Fii**), indicating that these changes depended on extracellular Ca^2+^, and were not merely time-dependent.

In addition to the general Ca^2+^-dependence of PPs, we were interested in where in the cell the PP-generating Ca^2+^ influx occurred. Recording with low-Ca^2+^ aCSF (0.1 mM Ca^2+^, 0.2 mM EGTA, 4 mM Mg^2+^) in the bath, high-Ca^2+^ aCSF (5 mM Ca^2+^, 0.5 mM Mg^2+^) was pressure- applied (“puffed”) locally, via a glass pipette, to either the perisomatic or the distal, apical dendritic part of the cell in the presence of 10 μM muscarine (**Fig 5A**). In low-Ca^2+^ aCSF, with the presence of muscarine, PPs were rarely induced by the spike train protocol, but when Ca^2+^ was locally applied to the perisomatic region (while the slice bath was still perfused with low-Ca^2+^), a PP was evoked regularly (**Fig 5Bi and 5D**).

However, when Ca^2+^ was locally applied to the distal apical dendrite, only much weaker PBD, usually without spiking, were elicited, and far less reliably (**Fig 5Bii and 5D**). Thus, the PBD was significantly larger with perisomatic Ca^2+^ application than with dendritic Ca^2+^ application (perisomatic Ca^2+^: 7.8 ± 2.4 mV, *n* = 8; dendritic Ca^2+^: 2.5 ± 2.0 mV, *n* = 9, *p* = 0.02). Also, the prevalence of spiking PPs was much higher after a perisomatic Ca^2+^ puff (**Fig 5D**), i.e. PPs were seen far more often after perisomatic than after apical dendritic Ca^2+^ application. However, there was no statistically significant difference in post-burst spiking between the perisomatic-puff and dendrite-puff groups, because one cell in the latter group showed a large, spiking PP, whilst none of the others in the group did (perisomatic Ca^2+^-puff: 27 ± 16 spikes, *n* = 8; dendritic Ca^2+^-puff: 22 ± 22 spikes, *n* = 9, *p* = 0.22) (**Fig 5Ci**). Again, there might have been technical difficulties with the local application in some cases.

Since we found that the PPs were reduced by blockers of TRPC4 and TRPC5 channels (**Fig 3**), which are known to be Ca^2+^-permeable (Owsianik et al., 2006), and we also found that the PPS were Ca^2+^-dependent (**Figs 4 and 5**), it seems likely that Ca^2+^ entry through TRPC4 and TRPC5 channels was involved in PP generation. However, since PPs have been demonstrated to be dependent on voltage-gated Ca^2+^ channels (VGCCs) in other cell types [12, 21, 38, 39], we sought to determine whether VGCCs could also play a role here.

We found that the non-specific VGCC blocker cadmium (Cd^2+^, 100 μM) suppressed PPs (**S3Ai Fig**), with large, significant reductions in both PBD (control: -1.8 ± 0.96 mV, *n* = 10; CdCl_2_: -10.9 ± 1.7 mV, *n* = 6, *p* < 0.01) (**S3Bi Fig**) and spikes (control: 5.4 ± 9.8 spikes, *n* = 10; CdCl_2_: -95 ± 12 spikes, *n* = 6, *p* < 0.01) relative to the control (**S3Bii Fig**).

Nickel (Ni^2+^), at 50 μM concentration, is known to block both T-type and R-type VGCCs [40]. We observed a range of effects of 50 μM Ni^2+^ on PPs (**S3Aii Fig**), but found no significant differences in either PBD (control: -1.8 ± 0.96 mV, *n* = 10; NiCl_2_: -1,4 ± 1.6 mV, *n* = 5, *p* = 0.77) (**S3Bi Fig**) or spiking (control: 5.4 ± 9.8 spikes, *n* = 10; NiCl_2_: -29 ± 16 spikes, *n* = 5, *p* = 0.11) compared to the control (**S3Bii Fig**). Wash-in of the L-type VGCC blocker nifedipine (10 μM; **S3Aiii Fig**) also caused no significant change in either PBD (control: -1.8 ± 0.96 mV, *n* = 10; nifedipine: -2.0 ± 0.88 mV, *n* = 5, *p* = 0.77) (**S3Bi Fig**) or spiking (control: 5.4 ± 9.8 spikes, *n* = 10; nifedipine: -18 ± 8.4 spikes, *n* = 5, *p* = 0.35) (**S3Bii Fig**). Similarly, the P-type VGCC blocker PD-173212 (10 μM; **S3Aiv Fig**) caused no significant changes in either PBD (control: -1.8 ± 0.96 mV, *n* = 10; PD-173212: -0.77 ± 1.1 mV, *n* = 7, *p* = 0.42)(**S3Bi Fig**) or spiking (control: 5.4 ± 9.8 spikes, *n* = 10; PD-173212: -10 ± 8.9 spikes, *n* = 7, *p* = 0.35) (**S3Bii Fig**).

Having established the importance of extracellular Ca^2+^ in generating PPs, we wished to investigate any possible involvement of intracellular Ca^2+^ stores. Intracellular Ca^2+^ release has been implicated in PPs [41, 42] and involved in the insertion of TRPC5 channels into the cell membrane and other TRPC channel activation (Tai et al., 2011), leading to PPs. mAChRs can induce Ca^2+^ release from intracellular stores via the αGq pathway, causing activation of IP3 receptors (IP3Rs) in the endoplasmic reticulum.

To test whether IP3Rs are involved in PP-generation in L2/3 pyramidal cells, we included the IP3Rs blocker heparin in the intracellular recording solution (5 mg/ml), and allowed 20 minutes after achieving whole-cell configuration, for it to diffuse into the cell (**S4 Fig)**. Heparin had no apparent effect prior to muscarine application. Thus, we found no difference in the PBD (control ICS: -0.33 ± 0.12 mV, *n* = 10; heparin ICS: -0.21 ± 0.25 mV, *n* = 4, *p* > 0.999) or spiking (control ICS: 0 ± 0 spikes, *n* = 10; heparin ICS: 0 ± 0 spikes, *n* = 4) between the control and heparin groups, suggesting that heparin did not affect basal properties. However, after 20 min. of heparin “dialysis”, application of muscarine did not induce robust PPs (**S4A Fig**), in sharp contrast to cells without i.c. heparin (**Figs 1–3; S4B, S4C Fig**). Thus, although most heparin-filled cells showed an AHP reduction and a small PBD (mean PBD increased) after muscarine was applied (**S4A and S4Ci Fig**), the changes were not statistically significant compared to time-matched recordings with control intracellular solution, neither for PBD (control: -0.21 ± 0.25 mV; muscarine: 1.1 ± 0.48 mV, *n* = 4, *p* = 0.25) (**S4Ci Fig**) nor spikes (control: 0 ± 0 spikes; muscarine: 0 ± 0.0 spikes, *n* = 4) (**S4Cii Fig**). However, the finding that intracellular heparin almost fully blocked the PP, suggests that Ca^2+^ release from intracellular stores is important for the generation of PPs in L2/3PCs.

### Plateau potentials could be generated in the absence of apical dendrites

Although plateau potentials have been found to depend on the apical dendrite in several cell types, and an apical dendritic origin is often assumed to be typical, there are reasons to think that PPs may also be generated in perisomatic or basal dendritic compartments in some cases. Thus, there is evidence that the TRPC channels that appear to be necessary for PPs in the L2/3PCs (**Fig 3**) are expressed only in the perisomatic compartment [43].

To test whether the PPs in the L2/3PCs are generated in or depend on the apical dendrite, we disconnected the apical dendrites from the perisomatic compartments by a scalpel blade cut through the slice between the L2/3PC somata and the pia (**Fig 6Ai**), to disconnect L1 and the superficial portion of L2 from the deep L2 and L3. Shortly (10–15 minutes) after the slice was cut, somatic whole-cell recordings were obtained, with Alexa- and biocytin-filled patch pipettes, from L2/3PC somata. During and after the recording, the Alexa-filled cells were observed immediately with fluorescence microscopy, and the biocytin-stained cells were later reconstructed, thus confirming that the distal part of the apical dendrite was indeed disconnected from the perisomatic compartments, at distances ranging from 20 to 260 μm (**Figs 6Aii and 6Aiii, 7**).

No clear differences in input resistance or resting membrane potential between truncated (cut) cells and control cells were found two minutes after break-in (**Tables 1 and 2**).

**Table 1:** Properties of neurons with truncated apical dendrites.

Cell number	Distance from pia (μm)	Remaining dendrite length (μm) a	RMP (mV)	Input resistance (MΩ)
1	610	160	-69.4	332.5
2	510	130	-68.5	114.3
3	490	150	-65.0	126.2
4	480	160	-75.4	149.8
5	600	260	-65.5	200.4
6	480	130	-75.8	155.0
7	580	170	-70.8	167.7
8	490	100	-72.7	111.6
9	500	90	-75.8	132.9
10	580	160	-66.6	159.6
11	430	60	-79.5	161.0
12	570	45	-69.4	139.6
13	350	40	-78.5	221.6
14	420	80	-70.3	220.6
15	500	20	-75.8	248.9
16	420	80	-77.3	211.4
17	480	120	-68.8	185.7
**Average**	**499.4 ± 17.3**	**115.0 ± 14.6**	**-72.1 ± 1.1**	**178.8 ± 13.7**

a Dendrite length measured using fluorescence or biocytin staining.

b Values are expressed as mean ± SEM.

**Table 2:** Properties of neurons with untruncated apical dendrites.

Cell number	Distance from pia (μm)	RMP (mV)	Input resistance (MΩ)
1	520	-75.7	156.7
2	560	-65.1	180.1
3	580	-67.6	157.2
4	490	-68.5	162.1
5	530	-68.9	159.5
6	630	-71.2	201.7
Average	551.7 ± 20.2	-69.5 ± 1.5	169.6 ± 7.3

a Dendrite length measured using fluorescence or biocytin staining.

b Values are expressed as mean ± SEM.

In the truncated cells, 10 μM muscarine induced PPs that appeared quite similar to those in intact cells (**Fig 6B**). Thus, most of the truncated cells showed PBDs that were significantly increased by muscarine (control: -0.29 ± 0.08 mV; muscarine: 7.2 ± 1.6 mV, *n* = 17, *p* < 0.01), but the muscarinic PPs/PBD amplitudes were smaller than in control cells (cut with muscarine: 7.2 ± 1.6 mV, *n* = 17; uncut with muscarine: 13.0 ± 1.5 mV, *n* = 6, *p* = 0.02) (**Fig 6Ci**). Muscarine also induced PP-evoked post-burst spiking in truncated cells (control: 0.1 ± 0.1 spikes; muscarine: 59 ± 16 spikes, *n* = 17, *p* < 0.01), with no significant difference between cut (truncated) and uncut cells (cut + muscarine: 59 ± 16 spikes, *n* = 17; uncut + muscarine: 83 ± 22 spikes, n = 6, *p* = 0.19) (**Fig 6Cii**).

Similar to uncut cells, truncated cells exhibited a range of PP types (**Fig 7**), but the PBD amplitude was not correlated with the remaining apical dendrite length (Spearman’s ρ = -0.18, *n* = 17; *p* = 0.48) (**Fig 6D**).

These results (**Fig 6**), combined with our results from local applications of Ca^2+^ (**Fig 5**), indicate that muscarinic PPs in L2/3PCs can be generated in the absence of the distal apical dendrite, thus probably being generated by perisomatic- and/or basal dendritic-dependent mechanisms.

### Optogenetic activation of cholinergic afferents

Having shown that bath-application of muscarine paired with a somatic spike-train can regularly induce spiking PPs in L2/3PCs, we wanted to investigate whether we could elicit spiking PPs in this cell type also by pairing somatic spike-trains with release of endogenous ACh from local cholinergic neurons and surviving cholinergic axons with presynaptic terminals in the slice (Obermayer et al. [44]. To do this, we used PFC brain slices from mice expressing both channelrhodopsin-2 (ChR2) and enhanced yellow fluorescent protein (EYFP) in cells expressing choline transferase (ChAT), i.e. cholinergic interneuron [45–47] (**S5A Fig**). After obtaining somatic whole-cell recording from an EYFP-labelled (i.e. cholinergic) neuron (n = 5), the slice was flashed with trains of brief (20 ms) light flashes at 25 Hz. Each flash reliably triggered an action potential in 4 of the 5 EYFP-labelled neurons tested (**S5B Fig**), thus demonstrating that the neurons were activated by the flashes.

Next, we tested the effect of light-induced release of endogenous ACh on whole-cell recorded L2/3PCs (*n* = 17). In these cells, a train of five or ten light flashes at 25 Hz, (similar to the protocol used by Obermayer et al [44] (**S5C Fig**), induced a small but significant PBD after a current-evoked burst of 7 spikes, but the PBD did not trigger spiking. Thus, there was a significant difference in post-burst membrane potential between the unpaired spike-trains and the spike-trains paired with light stimulation (unpaired: -0.15 ± 0.11 mV; with light stimulation: 0.69 ± 0.32 mV, *n* = 17, *p* = 0.01) (**S5D Fig**).

## Discussion

In this study, we describe a type of depolarising plateau potentials (PPs) in layer 2/3 pyramidal cells in rat prefrontal cortex (PFC) slices, following a train of action potentials evoked by somatic injection of brief current pulses, during perfusion with the metabotropic cholinergic agonist (mAChR) muscarine. To our knowledge this type of PP has not been described previously. Surprisingly, we found that unlike previously described PPs, which originate in the large apical dendrite of cortical pyramidal neurons, these L2/3 PPs are generated independently of the apical dendrite. Thus, the PPs persisted after we cut off the apical dendrite as close as ~50 μm from soma, and were sustained by local Ca^2+^ application only to the somatic and basal dendritic, but not apical, compartments.

The sustained spiking triggered by the PPs profoundly altered the input-output relationships of the L2/3PCs, and may thus may help trigger or sustain persistent network activity, suggested to underlie working memory, and reminiscent of “behavioural time scale synaptic plasticity” in hippocampal pyramidal cells [48]. This is of particular interest also because prefrontal L2/3PCs have been postulated to have a key role in consciousness, according to the Global Neuronal Workspace Theory of consciousness, as their long-range cortico-cortical connections provide the architecture required for the “global work-space", “ignition”, amplification, and sustained, reverberant activity, which are considered essential for conscious access [25, 26].

Persistent activity is believed to be enabled by several distinct mechanisms. This includes recurrent network connectivity allowing reentry of excitatory transmission to drive continued activity via feedback excitation, whilst also transmitting signals to more distal regions [49]. In addition to these connections, the regulation of synaptic weights can control the degree of recurrent activation of a generator network [50]. These could be called “synaptic” mechanisms of persistent network activity generation. On the other hand, plateau potentials maintain persistent spiking within an individual cell for seconds at a time or longer, and such persistent cellular activity could overcome the need for such fine tuned connectivity [51].The non-apical L2/3 PPs depended on metabotropic cholinergic (mAChR) or glutamatergic (mGluR) modulation, which is probably essential also for conscious experience, in both wakefulness and dreaming [6, 31, 52].

Pharmacological tests indicated that the non-apical L2/3 PPs depend on transient receptor potential (TRP) cation channels, both TRPC4 and TRPC5, and required external calcium (Ca^2+^) and internal Ca^2+^ stores, but not voltage-gated Ca^2+^ channels, unlike Ca^2+^-dependent PPs in other cortical pyramidal neurons. These L2/3 non-apical plateau potentials may be involved in prefrontal functions, such as access consciousness, working memory, and executive functions such as planning, decision-making, and outcome prediction [4, 23].

### Variable properties of muscarine-dependent plateau potentials

We examined the effect of muscarine on the membrane potential of PFC L2/3PCs following a train of action potentials. In control conditions, all cells tested exhibited a medium-duration (~0.2 s) afterhyperpolarisation (AHP). Application of 10 μM muscarine abolished the AHP and usually led to a long-lasting (~seconds) plateau potential (PP) that often triggered repetitive action potential firing. However, the PPs were variable. With 10 μM muscarine, 70% of PPs were accompanied by persistent firing (PF), i.e. spiking at a fairly constant rate that persisted for several seconds. Other PPs were self-terminating, with spiking that gradually slowed and stopped before the membrane potential returned to baseline; or there was only a large ADP with only a few, low-frequency action potentials. Finally, in around 30% of cells using 3 μM muscarine, only an ADP with no spiking was observed. This variability might be due to heterogeneity in the tested sample of cells, perhaps reflecting subsets of cells with different sets of channels or receptors [53–55]. The probability of generating a spiking PP was also greatly dependent on the concentration of muscarine used (**Fig 2C**).

### Evidence that plateau potentials depend on TRPC channels

Based on our results with various TRPC channel agonists and antagonists, we concluded that both TRPC4 and 5 were important for the generation of PPs.

TRPC5 currents can be activated or enhanced in different ways: intracellular signalling from G-protein coupled receptors can enhance TRPC currents via direct interaction with Gi proteins [56]; translocation of vesicular TRPC channels to the membrane leads to TRPC5 currents and PPs [57, 58]; and release of Ca^2+^ from intracellular stores increases TRPC current amplitudes through the channels interaction via STIM1 [59].

Whether or not TRPC channels underlie muscarinic ADPs and PPs may be highly cell-specific, even within specific brain regions. Thus, previous work has both found and failed to find evidence that TRPC5 channels contribute to muscarinic ADPs in various L5PCs in the PFC of rodents [34, 60]. Whilst our results seemed to show a greater effect on PPs using a TRPC5 antagonist than a TRPC4 antagonist, we did not test a range of concentrations, so the concentrations of TRPC4 and TRPC5 antagonists used might not have been strictly comparable. Therefore we cannot conclude which of these channel types is most important for the PPs in these cells, although both seem to contribute. Further work is needed to determine the exact contributions of different TRPC subtypes to these PPs in these cells.

### Evidence that plateau potentials depend on Ca^2+^ but not on voltage-gated Ca^2+^ channels

TRPC channels are activated by intracellular Ca^2+^ ions [61], which may enter the cell via channels in the plasma membrane or may be released from intracellular Ca^2+^ stores. Our observations that PPs were inhibited by Cd^2+^, but not by specific VGCC blockers, suggest that the PPs were generated by TRPC channels activated by intracellular Ca^2+^ that entered the cell via another route than VGCC.

Calcium release-activated channels (CRAC) channels such as ORAI channels have been shown to be blocked by Cd^2+^ with an IC50 of 200 μM [62], and ORAI channels and TRPC channels have been shown to associate in complexes [63]. Thus, the plateau potentials (PPs) may be generated by TRPC channels that form complexes with ORAI-based CRAC channels, whose Ca^2+^ influx activates the TRPC channels.

This mechanism of PP generation would be markedly different from that of the “typical” dendritic calcium-plateau/-spike in the distal apical hot zone in L5PCs, where strong depolarisation is required to activate L-type [21, 64] or R-type [20, 39] VGCCs. In L2/3PCs in the human neocortex, dendritic action potentials caused by coincident input have been shown to be Ca^2+^-based and often induce somatic spiking [7].

### Muscarinic plateau potentials in L2/3PCs are *non-apical*: They were not eliminated by truncation of the apical dendrites

PPs in cortical pyramidal cells have previously been suggested to be largely of dendritic origin, often triggered by convergent excitatory input to somatic and apical compartments [65]. There is also evidence that apical dendritic PPs in response to convergent excitatory input are important for both reportable sensory perception [9, 11] and in the overall alert brain state during normal wakefulness *in vivo* [9, 11], thus probably being involved in at least some forms of access consciousness [9, 11].

To test whether the muscarinic plateau potentials in L2/3PCs also depend on apical dendritic activity, we physically cut the apical dendrite at various distances from the soma. We found that the somatically recorded plateau potentials still persisted and triggered spiking (PF) even when most of the apical dendrite was disconnected (**Fig 6**). In addition, when the PPs were eliminated by perfusing the slice chamber with a low-Ca^2+^ aCSF, local application of Ca^2+^ only to the perisomatic region restored PP generation (**Fig 5**). Hence, it seems that these muscarinic plateau potentials are generated largely in the perisomatic and basal parts of the L2/3PCs including basal dendrites and soma (and possibly even parts of the axon, although the latter seems less likely). PPs evoked by basal dendritic input, and associated Ca^2+^ transients occurring in the basal dendrites, have been found previously in L5PCs in the prefrontal cortex [66]. However, it remains to be seen whether such PPs can be generated in vivo by natural basal synaptic input alone.

The well-known dendritic Ca^2+^ spikes/plateaus of L5PCs can typically be triggered by back-propagating Na^+^ action potentials originating in the soma, combined with local dendritic excitatory synaptic input. This activates VGCCs in the nexus of the apical trunk, the so-called Ca^2+^ hot zone, and the subsequent Ca^2+^ entry can trigger somatic/axonal action potential bursts [67]. This general mechanism may also occur in L2/3 cortical pyramidal cells. This study, however, demonstrates that the apical dendrite is not needed (at least not in its entirety) to initiate and maintain PPs and PF in some L2/3PCs.

### Consequences of amplification by plateau potentials in L2/3PCs

As described above, we found that cholinergic modulation caused L2/3PCs to greatly increase their spike output in response to a train of stimuli. The consequences of such amplification will be felt both locally and across large areas of the neocortex.

Thus, through the long-range cortico-cortical projections of L2/3PCs, the increased output during cholinergic influence will impact remote cortical targets. Within the PFC, L2 and L3PCs send axons to L5PCs [68], and specifically to cortico-thalamic PCs [69] which target thalamic nuclei, thereby completing a multi-synaptic reciprocal loop. Outside the PFC, L2 and L3PCs axons project primarily to other cortical areas, including the contralateral PFC, but also to subcortical areas such as the baso-lateral amygdala (BLA), forming direct reciprocal connections, and the striatum [70].

The origin of inputs to these cells may indicate which signals will be amplified by PPs in L2/3PCs. Layer 3 PCs receive their main excitatory input from the mediodorsal thalamus (MDT) [69], which is known to be important for attention and memory [71], with PFC-MDT connections being essential for object [72] and recency [73] recognition. Layer 2 PCs also receive input from the BLA [74]. BLA activity and reciprocal connections between the BLA and PFC have been shown to be important in encoding emotional states, and in particular emotionally weighted memories [75].

### Functional consequences of the non-apical origin of the plateau potentials

The non-apical origin of these PPs suggests that they preferentially will boost excitatory input of perisomatic and basal dendritic origin, as opposed to apical dendritic input, unlike the apical dendritic PPs and Ca^2+^-spikes typically observed in L5PCs [8]. Thus, the non-apical PPs of rat L2/3PCs seem poorly suited to perform the key type of coincidence detection and dendritic integration between two different streams of information that have been thoroughly characterised in rodent L5PCs [8, 10, 67, 76], and which forms the core of the dendritic integration theory of consciousness (DIT; [6]. An interesting, still open question is whether L2/3PCs in humans and other primates, whose apical dendrites are far longer and equipped with different ionic conductances than in rodents [77], and function more like rodent L5PCs [78] than rodent L2/3PCs in this respect. In other words: do human L2/3PCs have an apical dendritic PP mechanism that allows nonlinear integration between different information streams, like in rodent L5PCs?

### Effects of bath-applied muscarine vs. optogenetically released ACh

In most of our experiments, we used bath application of muscarine in an attempt to probe the muscarinic effects of ACh release. However, the effects of bath application may of course differ from those of physiological ACh release.

In vivo, ACh levels vary in both a tonic and transient manner, with low tonic levels throughout the brain during non-REM sleep, higher levels during REM sleep, and the highest during wakefulness [79]. During wakefulness, ACh release varies with behaviour [80], and the greatest transient increases have been shown to occur during working memory tasks and reward prediction [81]. Tonic changes in ACh levels associated with state transitions occur in all regions, though the level of change amongst cortical regions is heterogeneous [82]

Phasic changes associated with specific behavioural epochs appear to be largely coordinated between areas involved in a task, such as a working memory task requiring both the PFC and hippocampus [81]. Cholinergic stimulation also produces PPs in hippocampal neurons [12, 19]. That ACh levels co-vary between interconnected areas, the communication between which is crucial to a particular task, and that ACh produces PPs in cells in both areas could suggest the involvement of PPs in maintaining persistent activity and functional communication between the hippocampus and prefrontal cortex.

To better approximate natural ACh modulation, we activated cholinergic axons and terminals in the slice by optogenetics. This often caused a small, transient PBD, but no clear, persistent PPs or spiking. We do not know why the optogenetic method produced smaller PBD than bath-application, or whether the former effect was weaker (e.g. due to inferior function of the cholinergic axons and terminals in the slice) or the latter stronger than natural cholinergic modulation. However, our results do not seem to exclude the possibility that natural cholinergic modulation can induce PP generation in vivo, e.g. when combined with other modulators. Thus, although our optogenetic flash protocol evoked action potentials in the soma of local cholinergic interneurons (**S5B Fig**), we do not know if it efficiently triggered action potentials in long-range cholinergic axons and presynaptic terminals in the slice, axons that were cut off from the basal forebrain cholinergic nuclei that provide the majority of the ACh input to the PFC in vivo [83]. Perhaps many of the cut cholinergic axons were not excited or unable to release ACh, thus reducing the flash-induced release to a fraction of that occurring in vivo. In addition, cortical neurons in vivo are normally exposed to a cocktail of neuromodulators, which can sometimes have strongly synergistic effects. Several studies have shown that low concentrations of two neuromodulators applied together can induce PPs, even when one modulator alone has far less effect [19, 84]. In the PFC L2/3PCs, we found that also mGluR activation induced PPs. Therefore it is plausible that these cells can exhibit PPs in vivo when receiving natural, diverse neuromodulatory inputs. Experiments using combinations of agonists and optogenetically induced neuromodulator release, preferentially in vivo, are needed to further test these possibilities.

### Summary

We found that layer 2/3 pyramidal neurons in rat prefrontal cortex, when modulated by muscarinic cholinergic (mAChRs) input can generate plateau potentials (PPs) with sustained spiking, and that this can occur independently of distal apical dendrites.

The plateaus seem to depend on calcium, both external Ca^2+^ and internal Ca^2+^ stores, and on TRPC4 and TRPC5 cation channels, but not on voltage-gated Ca^2+^ channels. Since L2/3PCs have long-range cortical and subcortical connections, the increased, persistent spiking activity caused by muscarinic modulation of these cells, may have a widespread impact in brain states with high cholinergic input to the neocortex, such as during REM sleep and wakefulness.

## Supporting information

S1 Fig Plateau potentials can be induced by mGlu receptors. **A**—Example traces showing the PP induced by tACPD. **B**—Summary plots of the PBD (i) and spikes (ii) following the application of tACPD. Both PBD (i) and post-burst spiking (ii) change significantly after wash-in of tACPD (WSR test, *n* = 12).
(TIF)

S2 Fig Local FFA application at the soma and apical dendrite. **A**—Example traces showing the PP before and after local application of FFA (200 μM in the puffing pipette) aimed at the perisomatic region (i) or the apical dendrite (ii). **B**—Summary plots of the evoked PBD (i) and change in the number of evoked spikes (ii) before and after pressure-application of normal aCSF (Control) or FFA-containing aCSF aimed at the perisomatic region or apical dendrite. Although both the PBD and the evoked spike number were clearly reduced in some cells following application of FFA towards the perisomatic region, but not when applying FFA towards the dendrite, as illustrated in **Ai-ii**, and there were small changes in the mean values of these parameters across all cells tested only for FFA-applications towards the perisomatic region, not towards the dendrite (**Ai-ii**), the results varied considerably between cells, and neither the changes in PBD nor spikes weres found to be statistically significant across all the tested cells (MWU test, somatic control: *n* = 6; somatic FFA: *n* = 10; dendritic control: *n* = 3; somatic FFA: *n* = 5).
(TIF)

S3 Fig PPs are not prevented by VGCC blockers. **A—**Example traces of muscarinic PPs before and after application of VGCC blockers CdCl_2_(i), NiCl_2_ (ii), nifedipine (iii), and PD-173212 (iv). **B**—Summary plots of the change in PBD (i) and spikes (ii) after the application of different VGCC-blocking drugs. A significant change in PBD and post-burst spiking was observed after the wash-in of CdCl_2_, but not after the wash-in of the other VGCC blockers (MWU test, CdCl_2_: *n* = 6; NiCl_2_: *n* = 5; Nifedipine: *n* = 5; PD-173212: *n* = 7).
(TIF)

S4 Fig PPs are prevented by a block of IP3 receptors. **A**—Example traces of a cell dialysed with heparin, before and after application of muscarine. The difference in post-bust membrane potential expanded in the inset. **B**—Time course summary of PBD (i) and spikes (ii) following the application of muscarine in cells recorded with control intracellular solution (ICS) and heparin-containing ICS. **C**—Summary plots of the PBD (i) and spikes (ii) before and after the application of muscarine in cells recorded with control ICS and heparin ICS. With control ICS dialysing the cell, wash-in of muscarine significantly changed both PBD and post-burst spiking (WSR test, *n* = 10). When the cell is dialysed with heparin ICS, wash-in of muscarine does not generate spiking PPs, and no significant change in PBD is observed (WSR test, *n* = 4).
(TIF)

S5 Fig Optogenetic activation of a cholinergic neuron and afferents. **A**—High magnification photomicrograph of an EYFP-positive cell in L2/3 mPFC, with a patch pipette indicated by a black arrow. **B**—Recording from the EYFP-positive (presumably cholinergic) neuron seen in (A), showing the changes in membrane potential during a train of 10 brief flashes (20 ms) of blue light at 25 Hz. **C**—Example traces from an L2/3PC before and after a train of light flashes. The post-burst depolarisation before and after a flash of blue light is shown expanded in the inset. **D**—Summary plot of the PBD amplitude following a train of 10 brief flashes (like in B), presumably causing optogenetic activation of cholinergic afferents. A small, but significant PBD was observed between the control spike-train and spike-train paired with light stimulation (WSR, *n* = 17).
(TIF)

S1 Data Data associated with figures in the article. (XLSX)
