# Supplementary figures and images for: Non-apical plateau potentials and persistent firing induced by metabotropic cholinergic modulation in layer 2/3 pyramidal cells in the rat prefrontal cortex
Source: PLoS One. 2024 Dec 10;19(12):e0314652. doi: 10.1371/journal.pone.0314652 (PMC11630621; doi:10.1371/journal.pone.0314652)

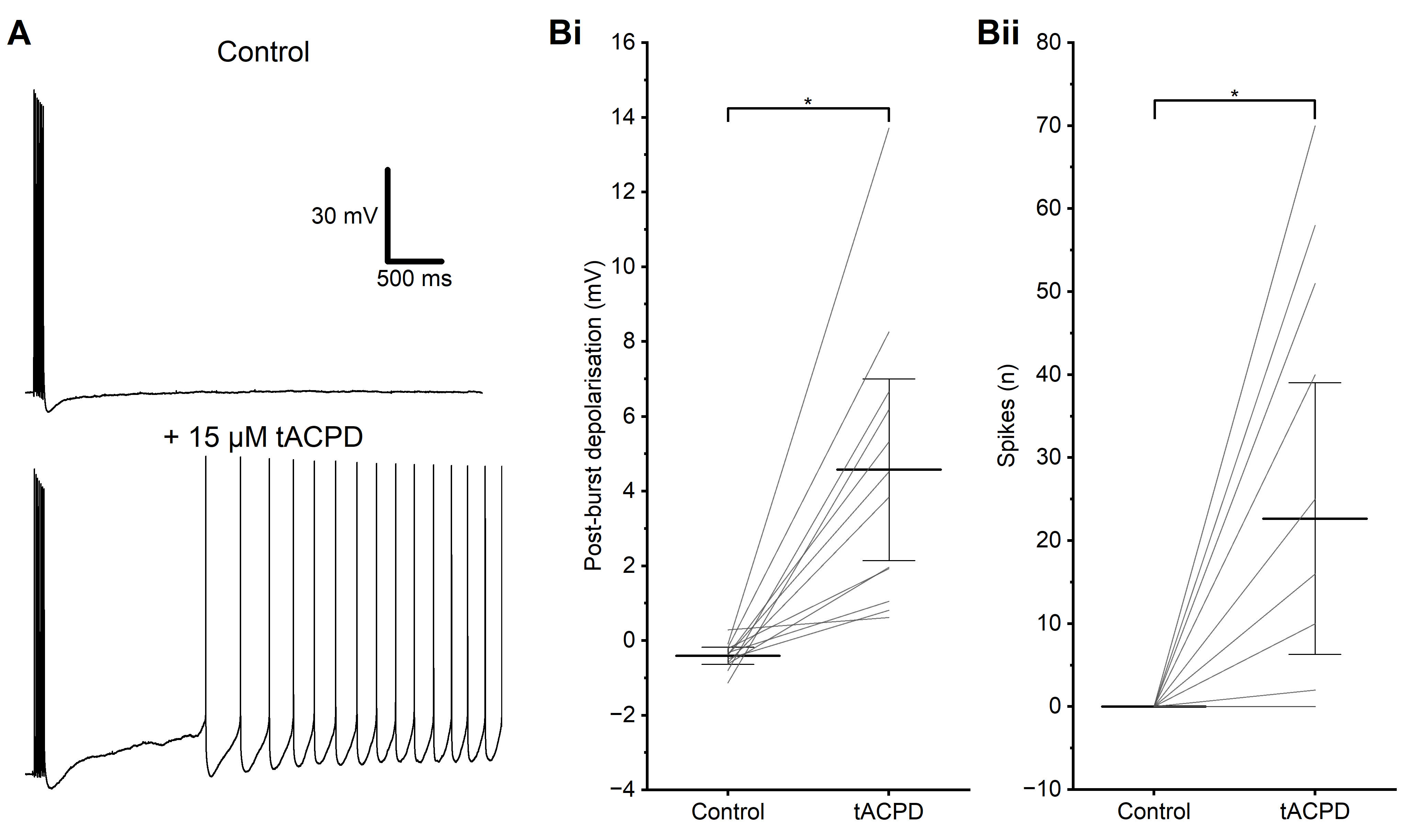

Supplement: S1 Fig — A—Example traces showing the PP induced by tACPD. B—Summary plots of the PBD (i) and spikes (ii) following the application of tACPD. Both PBD (i) and post-burst spiking (ii) change significantly after wash-in of tACPD (WSR test, n = 12). (TIF) [file pone.0314652.s001.tif]

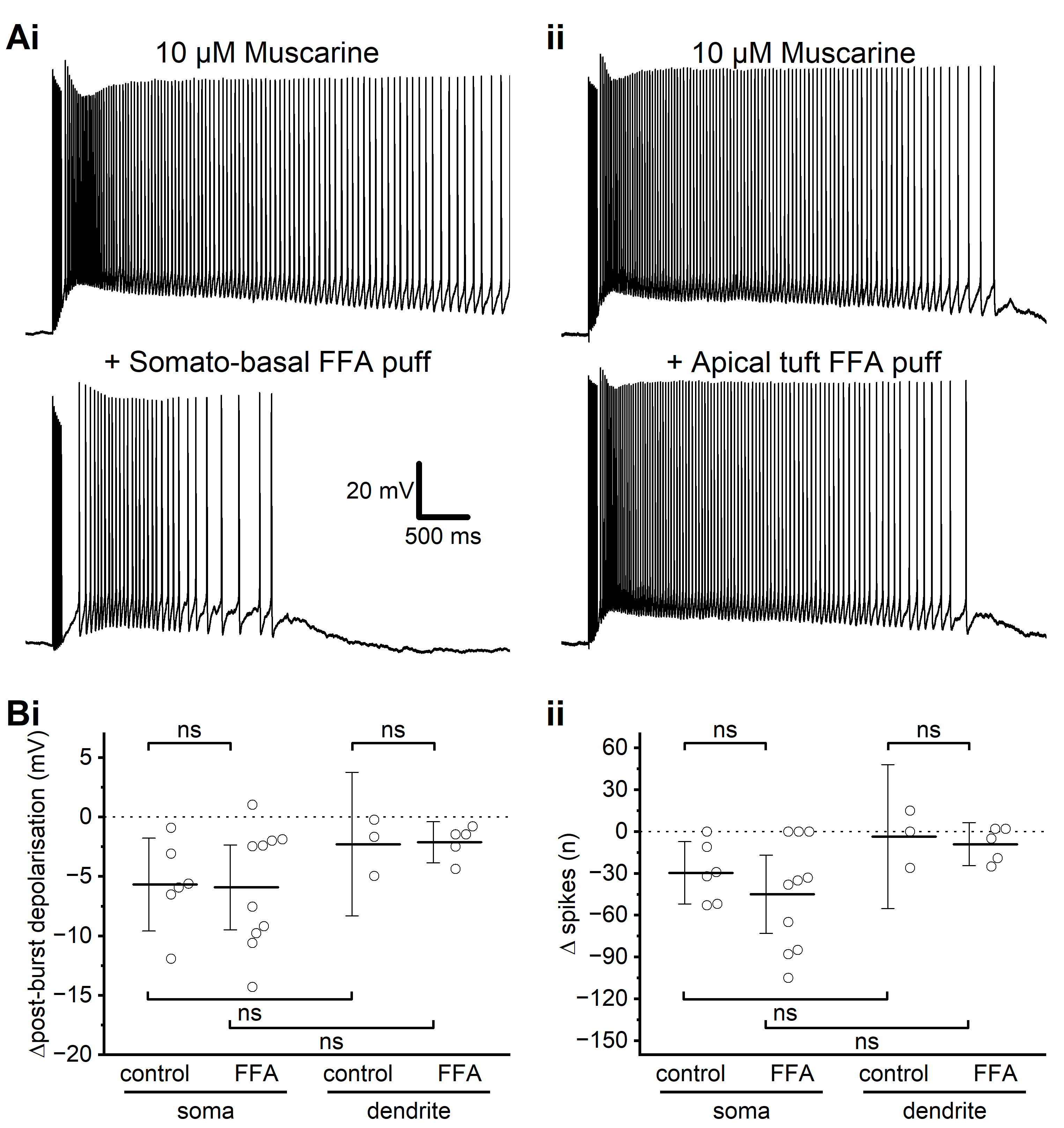

Supplement: S2 Fig — A—Example traces showing the PP before and after local application of FFA (200 μM in the puffing pipette) aimed at the perisomatic region (i) or the apical dendrite (ii). B—Summary plots of the evoked PBD (i) and change in the number of evoked spikes (ii) before and after pressure-application of normal aCSF (Control) or FFA-containing aCSF aimed at the perisomatic region or apical dendrite. Although both the PBD and the evoked spike number were clearly reduced in some cells following application of FFA towards the perisomatic region, but not when applying FFA towards the dendrite, as illustrated in Ai-ii, and there were small changes in the mean values of these parameters across all cells tested only for FFA-applications towards the perisomatic region, not towards the dendrite (Ai-ii), the results varied considerably between cells, and neither the changes in PBD nor spikes weres found to be statistically significant across all the tested cells (MWU test, somatic control: n = 6; somatic FFA: n = 10; dendritic control: n = 3; somatic FFA: n = 5). (TIF) [file pone.0314652.s002.tif]

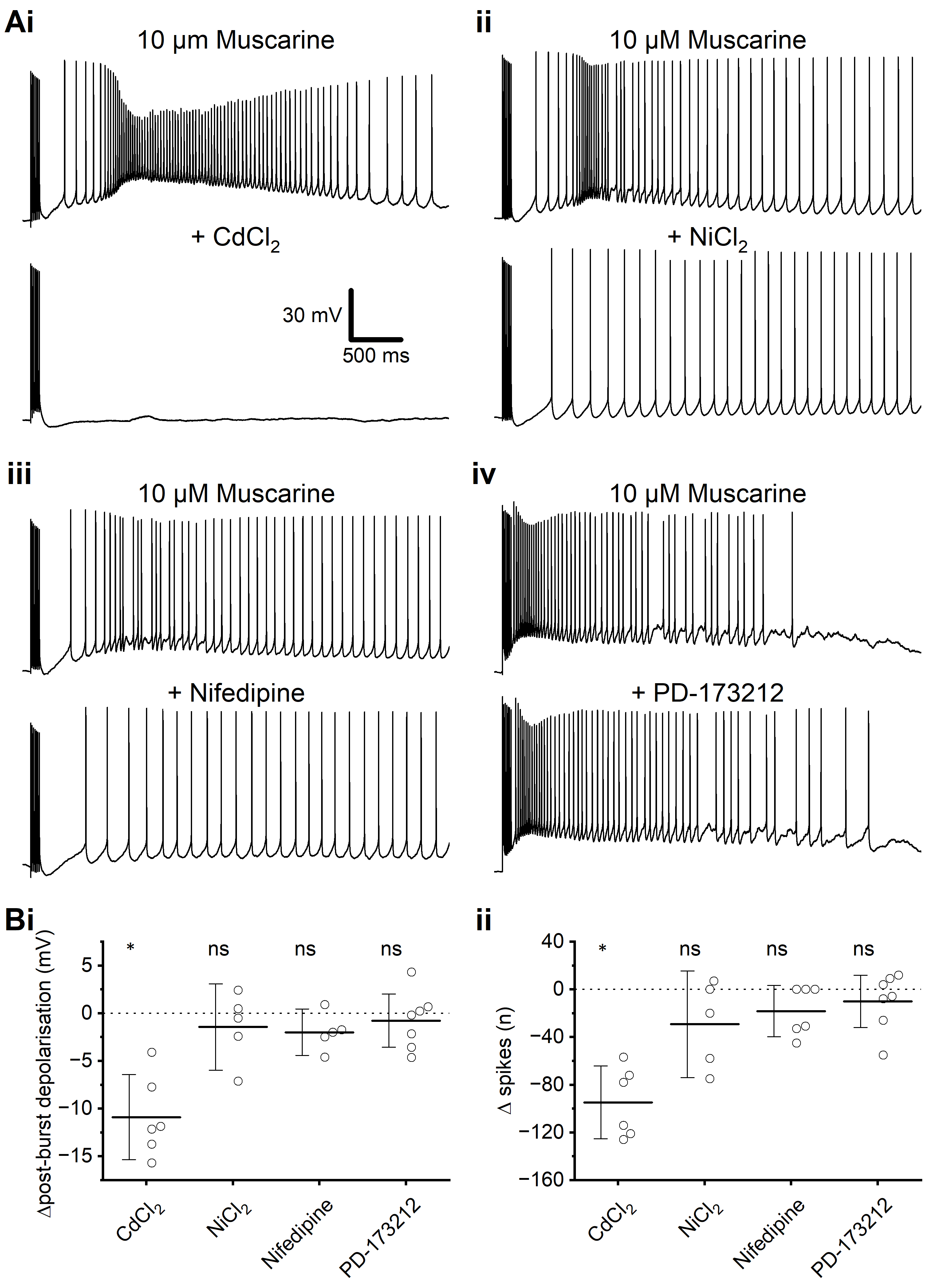

Supplement: S3 Fig — A—Example traces of muscarinic PPs before and after application of VGCC blockers CdCl2(i), NiCl2 (ii), nifedipine (iii), and PD-173212 (iv). B—Summary plots of the change in PBD (i) and spikes (ii) after the application of different VGCC-blocking drugs. A significant change in PBD and post-burst spiking was observed after the wash-in of CdCl2, but not after the wash-in of the other VGCC blockers (MWU test, CdCl2: n = 6; NiCl2: n = 5; Nifedipine: n = 5; PD-173212: n = 7). (TIF) [file pone.0314652.s003.tif]

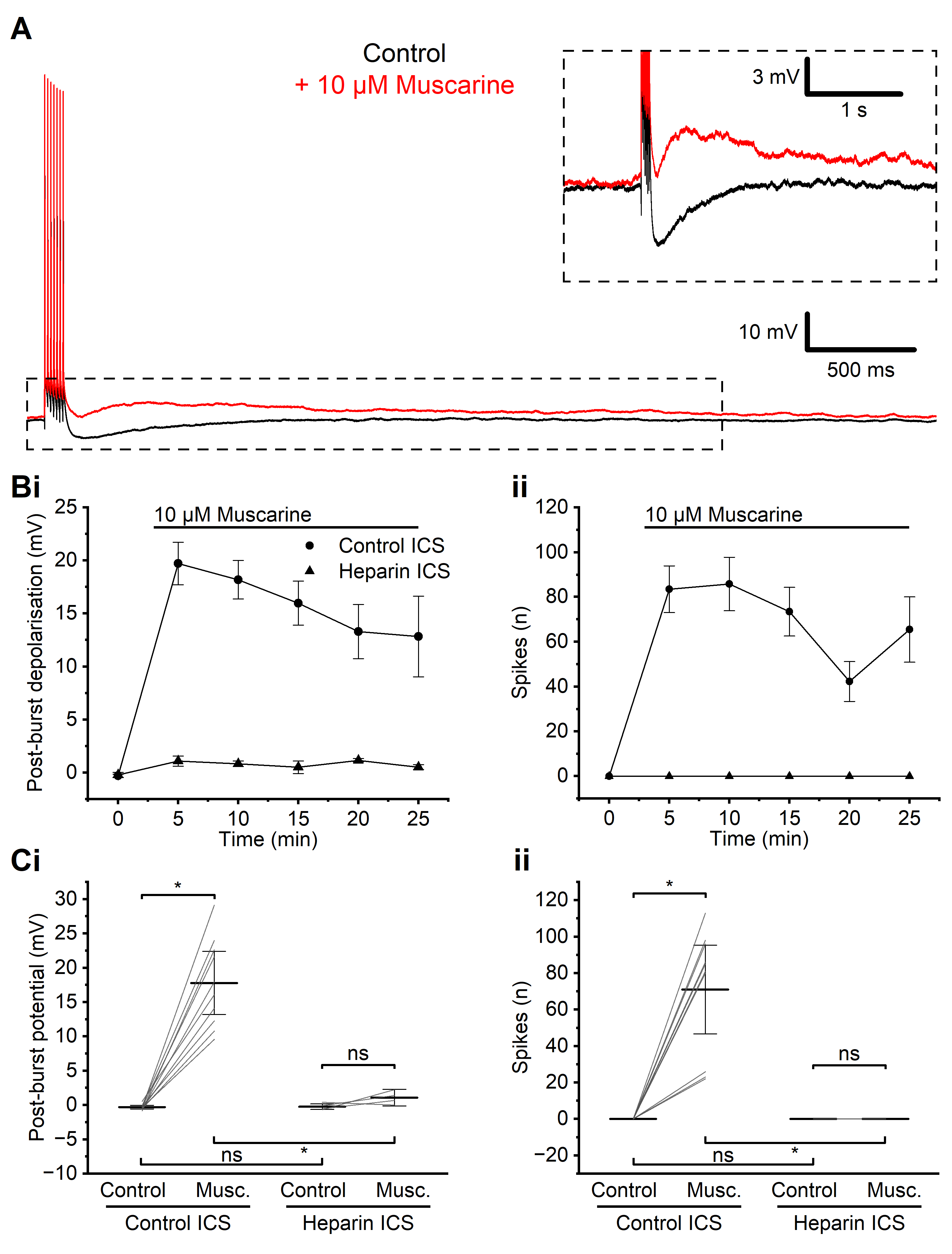

Supplement: S4 Fig — A—Example traces of a cell dialysed with heparin, before and after application of muscarine. The difference in post-bust membrane potential expanded in the inset. B—Time course summary of PBD (i) and spikes (ii) following the application of muscarine in cells recorded with control intracellular solution (ICS) and heparin-containing ICS. C—Summary plots of the PBD (i) and spikes (ii) before and after the application of muscarine in cells recorded with control ICS and heparin ICS. With control ICS dialysing the cell, wash-in of muscarine significantly changed both PBD and post-burst spiking (WSR test, n = 10). When the cell is dialysed with heparin ICS, wash-in of muscarine does not generate spiking PPs, and no significant change in PBD is observed (WSR test, n = 4). (TIF) [file pone.0314652.s004.tif]

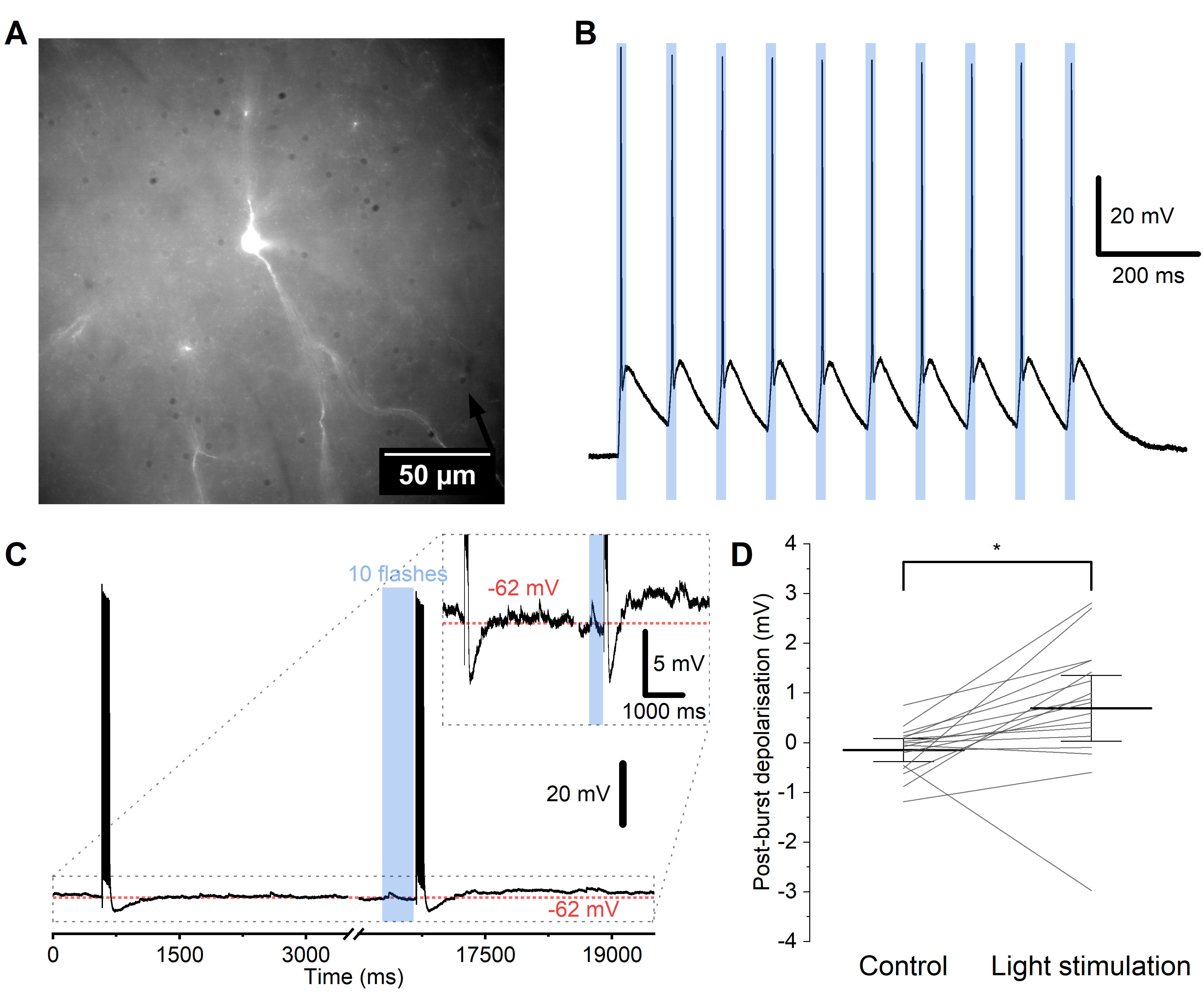

Supplement: S5 Fig — A—High magnification photomicrograph of an EYFP-positive cell in L2/3 mPFC, with a patch pipette indicated by a black arrow. B—Recording from the EYFP-positive (presumably cholinergic) neuron seen in (A), showing the changes in membrane potential during a train of 10 brief flashes (20 ms) of blue light at 25 Hz. C—Example traces from an L2/3PC before and after a train of light flashes. The post-burst depolarisation before and after a flash of blue light is shown expanded in the inset. D—Summary plot of the PBD amplitude following a train of 10 brief flashes (like in B), presumably causing optogenetic activation of cholinergic afferents. A small, but significant PBD was observed between the control spike-train and spike-train paired with light stimulation (WSR, n = 17). (TIF) [file pone.0314652.s005.tif]
